# The Second Nitrogen Fits: Development of Diazine-Based Macrocyclic Chelators to Stably Bind Radionuclides Used in Targeted Alpha Therapy

**DOI:** 10.1021/acs.inorgchem.6c01646

**Published:** 2026-09-16

**Authors:** Magdalena K. Blei, Lukas Waurick, Björn Drobot, Santiago A. Brühlmann, Charlotte Duchemin, Edgars Mamis, Muhammad Inzamam, Thierry Stora, Nadia Audouin, Thomas Sounalet, Klaus Kopka, Thorsten Stumpf, Sven Stadlbauer, Jerome Kretzschmar, Constantin Mamat

**Affiliations:** † 28414 Helmholtz-Zentrum Dresden-Rossendorf, Institute of Radiopharmaceutical Cancer Research, Bautzner Landstr. 400, D-01328 Dresden, Germany; ‡ TU Dresden, School of Science, Faculty of Chemistry and Food Chemistry, D-01062 Dresden, Germany; § Helmholtz-Zentrum Dresden-Rossendorf, Institute of Resource Ecology, Bautzner Landstr. 400, D-0132 Dresden, Germany; ∥ 61779 CERN, The European Organization for Nuclear Research, CH-1211 Geneva, Switzerland; ⊥ GIP ARRONAX, 1 Rue Aronnax, 44800 Saint-Herblain, France; # Subatech, UMR 6457, 30531 IMT Atlantique, CNRS/IN2P3, Nantes Université, 44307 Nantes, France; ∇ National Center for Tumor Diseases (NCT), NCT/UCC Dresden, a partnership between DKFZ, University Hospital Carl Gustav Carus and Faculty of Medicine at TU Dresden, and Helmholtz-Zentrum Dresden-Rossendorf (HZDR), D-01307 Dresden, Germany; ○ German Cancer Consortium (DKTK), Partner Site Dresden, Fetscherstr. 74, D-01307 Dresden, Germany

## Abstract

Identifying suitable chelators for ^212^Pb, ^223^Ra, and ^225^Ac and their diagnostic counterparts ^131^Ba, ^133^La, and ^203^Pb remains one of the major challenges in the development of radiopharmaceuticals for targeted alpha therapy. Four novel macrocyclic chelators containing the 2,4- and 2,6-pyrimidine moieties (**pym-2,4** and **pym-2,6**), along with a combination thereof (**pym-mix**), as well as the pyrazine moiety (**pazi**), were synthesized and fully characterized. Protonation constants (p*K*_a_) were determined using ^1^H NMR spectroscopy. Stability constants (log *K*) for the complexes with La^3+^ (11.8–12.6), Ba^2+^ (10.1–10.3), and Pb^2+^ (15.3–15.5) are lower compared to macropa. However, pH-dependent binding affinities (p*K*_d,M_) with the novel chelators are higher for Ba^2+^ (8.7–8.9) and comparable or slightly lower for La^3+^ and Pb^2+^ at pH 6. DFT calculations enabled comprehensive elucidation of the molecular structures, facilitating in-depth analyses of the coordination environment. The determined ^15^N NMR chemical shifts of free chelators and complexes help to explain distinct complexation behavior. Finally, the novel chelators were radiolabeled with ^131^Ba, ^223^Ra, ^133^La, ^225^Ac, ^203^Pb, and ^212^Pb, and the radiochemical conversions were compared to results with macropa. All ^223^Ra complexes were incubated in human serum, pointing out an increased kinetic inertness and stability.

## Graphical abstract

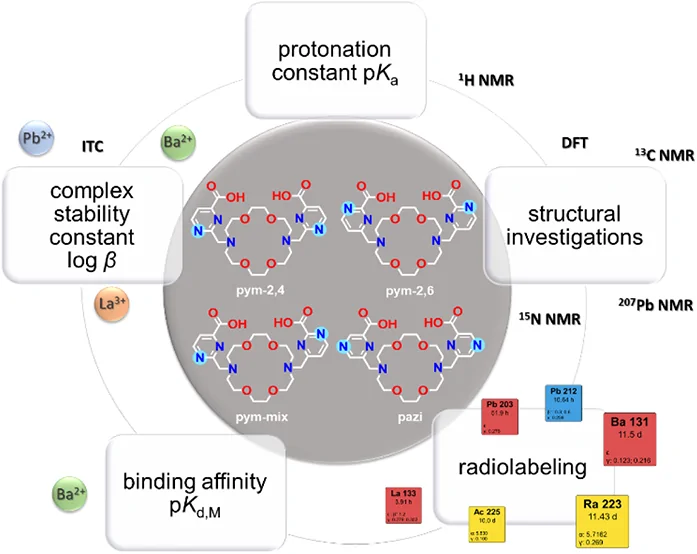

## Introduction

In nuclear medicine, the radiopharmaceutical theranostic concept has gained increasing importance, particularly in the development of radiopharmaceuticals^1^ for targeted ligand therapies especially in oncological applications.^2,3^ It combines radionuclide-based imaging using β^+^ emitters for Positron Emission Tomography (PET) or γ emitters for Single Photon Emission Computed Tomography (SPECT) with radionuclide therapy employing α, β^–^, or Auger electron-emitting radionuclides.^4,5^ Theranostic radiopharmaceuticals typically consist of a biological or pharmacological vector molecule, which specifically binds to a biological target highly upregulated in malignant tissues. This vector is stably conjugated via a linker or spacer to a chelating unit or prosthetic group that enables the stable coordination of the metallic radionuclide.^6,7^ In this regard, the search for radiometals as well as the design of chelating systems to create stable radiometal complexes continue to be an important criterion of radiopharmaceutical sciences.^5,8^

The specific nuclear properties of the used radionuclides enormously influence the biological effect and therefore the therapeutic outcome, which is mostly dependent on the path length and the linear energy transfer (LET) of the emitted particles. Targeted alpha therapy (TAT) focuses on α emitters having a high LET of about 100 keV/μm and a path length of around 50–100 μm, which leads to a high ionization potential.^9^ The emitted α particles directly impact the DNA by inducing double strand breaks with a higher cytotoxicity than β^–^ emitters.^10^ Furthermore, the short path length is advantageous to reduce the irradiation of the surrounding healthy tissue, which improves the treatment, especially for smaller tumors, metastases, or isolated cancer cells.^3,11,12^

Even though there are multiple α emitting radionuclides (>300) known, only a few of them show the required properties for clinical applications, which were summarized by Eychenne *et al*.^13^ as “the hopeful eight”: ^225^Ac, ^211^At, ^212^Bi, ^213^Bi, ^212^Pb (β-emitter), ^223^Ra, (^224^Ra), ^227^Th, and ^149^Tb. However, within the context of developing a radiometal conjugate, it is imperative to ensure the stable binding of radiometals by a chelator. A selection of important chelators suitable for relevant short-lived α-particle emitting radiometals are shown in Figure 1.

**Figure 1:**
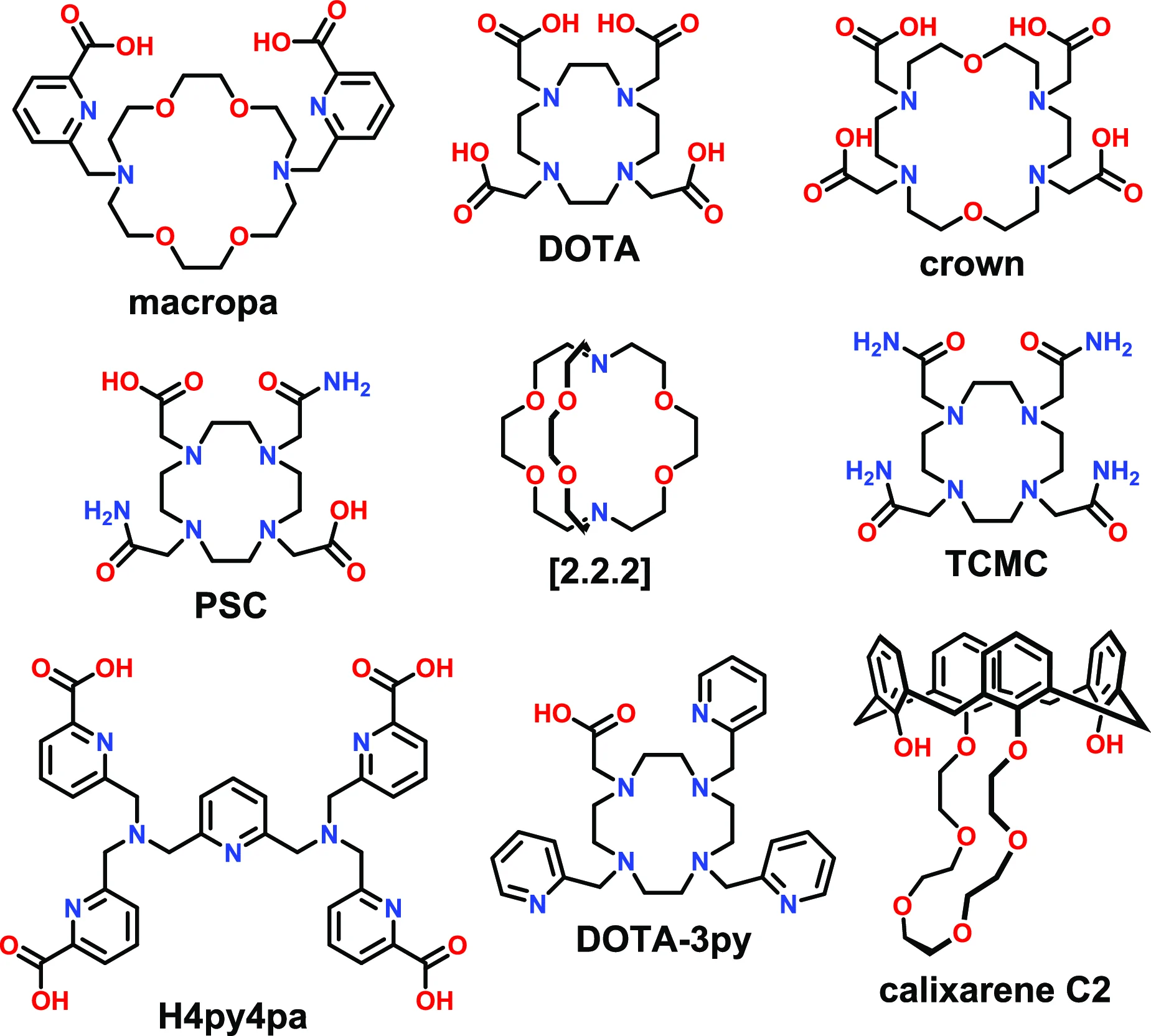
A selection of relevant and promising chelators used for the stable coordination of α-particle emitting radiometals ^225^Ac, ^212^Bi, ^223/224^Ra, and ^212^Pb (β-emitter).

With a total of four α decays, ^225^Ac (*t*_1/2_ = 9.9 d, E_α_ = 9.53 MeV) can efficiently be used for cancer treatment in nuclear medicine.^14^ In addition to DOTA, macropa^15–18^ has proven its worth by being an excellent chelating agent and is able to form a highly stable ^225^Ac-complex under mild conditions in a short period of time. Nevertheless, the search for a diagnostic counterpart^19^ for this combination is still ongoing and one promising candidate is the β^+^ emitter ^133^La (*t*_1/2_ = 4.8 h) for PET imaging.^20–22^ ^212^Pb (*t*_1/2_ = 10.6 h), per se a β^–^ emitter, is of particular interest in the context of TAT,^23^ as it functions as an *in vivo* generator of its first progeny ^212^Bi (*t*_1/2_ = 60.6 min),^24^ which is an α emitter. Together with ^203^Pb (*t*_1/2_ = 51.9 h, γ emitter), which can be used as diagnostic counterpart in SPECT imaging, ^212^Pb and ^203^Pb form an ideal true matched theranostic pair.^25^ While DOTA showed promising results for complexing Pb^2+^,^26^ there were also some shortcomings that needed improvement,^27,28^ leading to the development of new cyclen-based chelators: TCMC,^29^ PSC,^30^ and DOTA-3py^31^ (see Figure 1). Notably, macropa^32^ showed a high level of radiochemical conversion with ^212^Pb under mild radiolabeling conditions combined with a high stability of the resulting Pb-complexes.

Both radium isotopes ^223^Ra and ^224^Ra are also promising candidates for TAT^33^ because of their decay properties such as their physical half-lives (^223^Ra: *t*_1/2_ = 11.4 d, ^224^Ra: *t*_1/2_ = 3.9 d) as well as multiple alpha decays with progenies having short half-lives.^34^ However, for an application in a radionuclide conjugate, the stable binding of Ra^2+^ to a chelator needs to be ensured and finding chelators for Ra^2+^ is a big challenge.^35–38^ In previous studies it was shown that the alkaline earth radiometal ^131^Ba (*t*_1/2_ = 11.5 d) as diagnostic radionuclide for SPECT imaging^39^ is a well-matched pair with radium isotopes forming isostructural and isoelectronic radioconjugates with Ra^2+^ and Ba^2+^ having a similar biological behavior.^40^ Nevertheless, previously tested chelators based on polyaminopoly­(carboxylic acids)^41,42^ such as DOTA^43^ were inefficient in complexation and not stable *in vivo*. Therefore, novel chelators like calixarenes,^44–46^ and aza-crown ethers^47,48^ were investigated, showing an improved behavior, but were still not efficient enough for TAT applications due to the still insufficient stability *in vivo* of the formed complexes. To our knowledge, the most convincing results were achieved with macropa.^49^ Recently, *in vivo* studies were performed in healthy mice using ^131^Ba, as free [^131^Ba]­Ba^2+^ or as ^131^Ba-macropa complex.^39^ Biodistribution experiments revealed the expected rapid bone uptake of free [^131^Ba]­Ba^2+^, whereas [^131^Ba]­[Ba­(macropa)] showed a fast clearance from the blood, thereby showing a significantly lower accumulation in the bones, but further optimization may be beneficial. Although these findings were confirmed by Abou *et al.* ^50^ in their first *in vivo* studies with [^223^Ra]­[Ra­(macropa)] and conjugates, the identification of suitable chelating systems for radium remains a major challenge for radiopharmaceutical applications of this radiometal.^51^

In order to shed light on this obstacle, four novel macrocyclic chelators containing diazine carboxylate functions were synthesized and fully characterized by NMR spectroscopy and MS. Their protonation constants (p*K*_a_) and thermodynamic stability constants (log *K*) for complexes with La^3+^, Ba^2+^, and Pb^2+^ were determined. The binding affinities (p*K*_d,M_) for Ba^2+^ were calculated as a function of pH and compared with already known chelator macropa.

In general, N-heterocyclic 6-membered aromatic rings are electron poor having an influence on the p*K*_a_ of any appended carboxylic group. The introduction of an additional aromatic nitrogen atom further influences electron delocalization effects at those carboxylate groups, and can be dependent on the position of the respective second nitrogen. This impacts the coordination chemistry and properties of the chelator, especially for the group 2 metal ions: we hypothesize, it will lead to highly stable complexation of group 2 metal ions, as well as other metal ions. Therefore, we have prepared and tested a library of regioisomeric diazines to examine the best position of the second nitrogen, and assess suitability for use with important alpha-emitting radiometal ions. The detailed elucidation of the molecular structure was carried out by a combination of NMR experiments and computational methods. The complexation of appropriate metals for targeted alpha therapy (^212^Pb, ^223^Ra, ^225^Ac) and their diagnostic counterparts (^203^Pb, ^131^Ba, ^133^La) was investigated and compared with the respective radiometal-macropa results. Furthermore, the kinetic inertness of the obtained novel ^223^Ra-labeled complexes was tested in human serum.

## Results and Discussion

Especially for both therapeutic radionuclides ^223^Ra and ^224^Ra, but also for the diagnostic counterpart ^131^Ba, there is still a gap in suitable chelating systems to stably bind these radionuclides. Macropa shows one of the highest complex stabilities with the heavy group 2 metal ions, however the complex stability is still insufficient leading to a release of the cation in biological environments. To overcome this situation, the side arms of macropa were modified by introducing an additional nitrogen atom leading to different diazine carboxylate moieties.

### Synthesis of the Chelators

Starting from the methylesters of methyldiazinecarboxylates, the chelators were synthesized over three steps. To achieve this, three modified side arms **2a**–**c** ^52^ containing the diazine moiety were prepared by the radical bromination of the respective methyl methyldiazine carboxylates.

To connect the new side arms to the diazamacrocycle, Kryptofix 2.2 (**1**) was reacted with the respective methyl (bromomethyl)­diazinecarboxylates **2a**–**c** in excess in the presence of diisopropylethylamine as base in acetonitrile and stirred at 75 °C overnight. The desired compounds **3a**–**c** were obtained in yields of 65–79% after purification using column chromatography. To saponify the ester functions in the last step, the compounds **3a**–**c** and lithium hydroxide (6 equiv) were dissolved in a 1:1 methanol/water solution and stirred at 50 °C overnight. After purification by semi-preparative HPLC, the desired chelators **pym-2,4**, **pym-2,6**, and **pazi** were obtained in yields of 28–92% as colorless solids. The three-step synthesis path is displayed in Figure 2.

**Figure 2:**
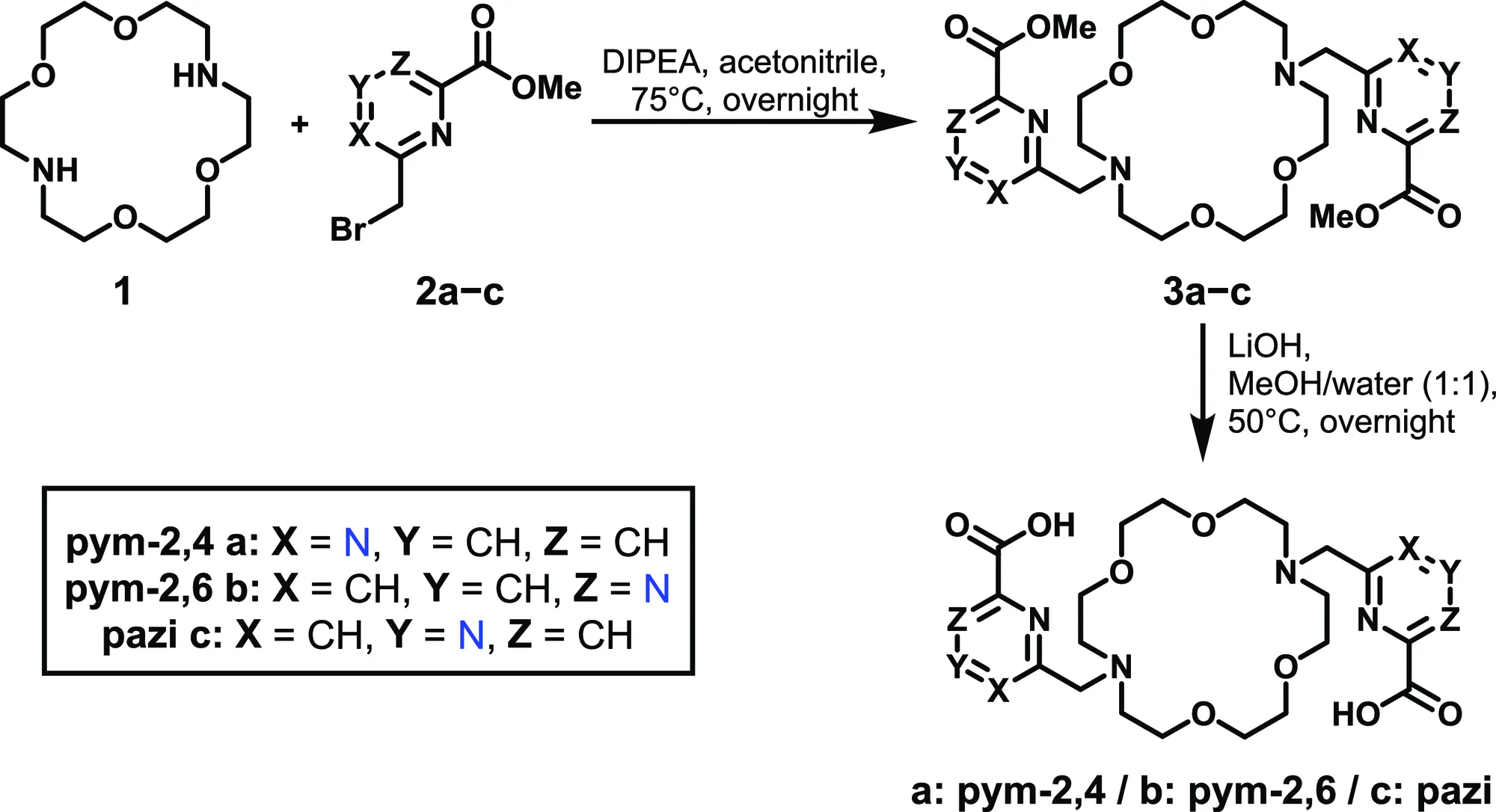
General synthesis of the novel chelators **pym-2,4**, **pym-2,6**, **pym-mix**, and **pazi**.

The synthesis of the mixed chelator **pym-mix** follows a slightly different procedure. First, Kryptofix 2.2 (**1**) was reacted with only 1 eq. of **2b** in high dilution to ensure the alkylation on only one of the secondary amine moieties of **1**. After purification, the intermediate was reacted with the second side arm **2a**. Finally, the methyl groups of the ester functions in **3d** were cleaved under strongly basic conditions with LiOH in a methanol/water mixture to obtain **pym-mix**.

A comprehensive characterization of the four novel chelators **pym-2,4**, **pym-2,6**, **pym-mix**, and **pazi** displayed in Figure 3 was performed by ^1^H, ^13^C, ^15^N NMR, ^1^H,^1^H–COSY, ROESY, ^1^H,^13^C-HSQC as well as ^1^H,^13^C- and ^1^H,^15^N-HMBC using a 20 mM D_2_O solution of each ligand at pD 4. A summary of corresponding analytical data for all four novel chelators is compiled in the SI.

**Figure 3:**
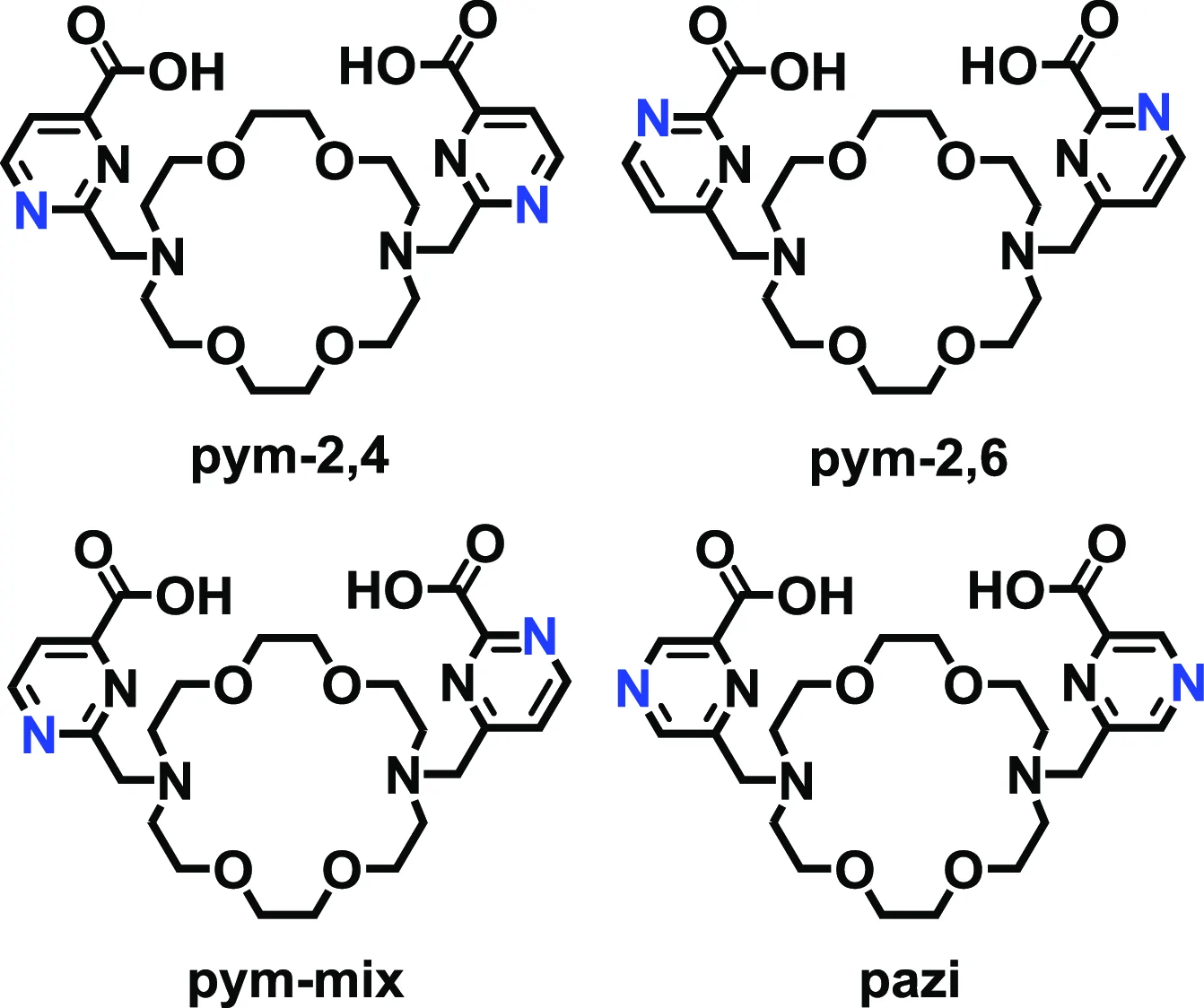
Chemical structures of the four novel chelators **pym-2,4**, **pym-2,6**, **pym-mix**, and **pazi**. The additional nitrogen atom at the specific position in the aromatic ring is highlighted in blue.

### Determination of Thermodynamic Equilibrium Constants

As described for macropa by Blei *et al.*,^32^ p*K*_a_ values (Table 1) were obtained from ^1^H NMR pD-titration series, allowing for the assessment of the pH range in subsequent ITC and radiolabeling experiments. Therefore, pD-titration samples with a chelator concentration of 1 mM were prepared using a 0.1 M NaCl-D_2_O solution, covering a pD range from 0.7 up to 11.3, with increments of approx. 0.2 pD units. With an increasing pH, a successive chelator deprotonation is observed, resulting in a general upfield-shift of the signals (cf. Figure S1 of SI), which appears due to the higher electron density remaining on the chelator upon removal of positive charges (H^+^). Two of the protons are abstracted from the carboxyl groups in the acidic range and two more from the nitrogen atoms of the macrocyclic ring amines (ammonium form) under near-neutral to alkaline conditions. The variable line widths (signal width at half amplitude) in the ^1^H NMR spectra are meaningful indicators of kinetic processes associated with the protonation/deprotonation regime. In particular, the signals of ^1^H nuclei in direct vicinity of the amino groups show a significant broadening in the pH range close to the corresponding p*K*_a_ values.

**Table 1:** Deuterium-Corrected p*K*_a_ Values^a^ of the Chelators **pym-2,4**, **pym-2,6**, **pym-mix**, and **pazi** Obtained from ^1^H NMR Spectroscopy at an Ionic Strength of *I* = 0.1 M NaCl in Comparison to Reported Data for macropa^32^

	**p** *K*_**a,1**_	**p** *K*_**a,2**_	**p** *K*_**a,3**_	**p** *K*_**a,4**_
Chelator	H_4_L^2+^→ H_3_L^+^	H_3_L^+^→ H_2_L^0^	H_2_L^0^→ HL^–^	HL^–^→ L^2–^
macropa^32^	2.40	3.13	6.80	7.73
**pym-2,4**	1.23	1.85	6.13	6.68
**pym-2,6**	1.25	2.00	5.94	6.95
**pym-mix**	1.57	1.77	6.03	6.95
**pazi**	1.44	2.19	5.82	7.00

a Estimated error values of ±0.15, considering uncertainties arising from pH electrode uncertainty including temperature effects during calibration and measurement as well as varying exchangeable H contents arising from the protonated chelator starting material and water of crystallization.

The stability constants log *K* of the formed complexes as well as the binding affinities p*K*_d,M_ of the four novel chelators using stable isotopes of corresponding metals Pb^2+^, La^3+^, and Ba^2+^ were determined by means of isothermal titration calorimetry (ITC). An exceptionally high binding affinity makes measurements at neutral pH unsuitable for evaluation. The effective saturation of the chelator by the metal is not suitable for the direct determination of complex formation constants as it does not allow for a useful measurement of the equilibrium free metal concentration required for the law of mass action. By considering the p*K*_a_ values of the coordinating groups, the pH was lowered to moderate the affinity and to enable a reliable determination of the thermodynamic parameters.^32^ Accordingly, the pH was set to 2 for Pb^2+^ and to 5 for La^3+^ and Ba^2+^. As with macropa, an endothermic reaction was observed for the La^3+^ complexation, while an exothermic reaction was observed for the other two metal ions Pb^2+^ and Ba^2+^ during complexation. This behavior has been repeatedly observed and is ascribed to a relatively higher energy required to replace coordinating water molecules from the trivalent La^3+^ ion, as compared to the divalent Pb^2+^ and Ba^2+^ ions.^32,53,54^ Furthermore, a small increase was observed in the beginning of the measurement for the complexation with La^3+^. To investigate whether deprotonation/protonation of the macropa ligand during the titration or formation of an intermediate 2:1 La-chelator-complex was responsible for the observed behavior, the measurements were repeated in MOPSO buffer (pH 6.6). The initial increase at the beginning of the titration was not observed in this MOPSO-buffered system, suggesting that the effect is due to the former reason. Thus, an evaluation model for the La–macropa system was chosen, identical to the other metal systems, assuming a single complex with the expected 1:1 stoichiometry. By taking advantage of the p*K*_a_ values determined by NMR spectroscopy, pH-independent complex stability constants (log *K*) were obtained from the ITC titrations for the various new macropa-based chelators across the different metal ions (Table 2).

**Table 2:** The Complex Stability Constants log *K* and the Binding Affinities p*K*_d,M_ at pH 6 for the Respective Complexes Composed of the Chelators **pym-2,4**, **pym-2,6**, **pym-mix**, and **pazi** with Pb^2+^, La^3+^, and Ba^2+^ Compared with Data Reported for Macropa at an Ionic Strength of *I* = 0.1 M NaCl^32^

M^n+^	complex	n	log *K*	p*K*_d,M_ (pH = 6)
Pb^2+^	[Pb(macropa)]	1.02 ± 0.01	18.5 ± 0.01	15.7
	[Pb(pym-2,4)]	0.96 ± 0.02	15.5 ± 0.16	14.2
	[Pb(pym-2,6)]	0.86 ± 0.03	15.3 ± 0.16	13.9
	[Pb(pym-mix)]	1.09 ± 0.04	15.5 ± 0.06	14.2
	[Pb(pazi)]	1.09± 0.04	15.4 ± 0.22	14.0
La^3+^	[La(macropa)]^+^	0.86 ± 0.03	13.9 ± 0.19	11.1
	[La(pym-2,4)]^+^	0.91 ± 0.05	11.8 ± 0.17	10.5
	[La(pym-2,6)]^+^	0.97 ± 0.07	12.6 ± 0.38	11.2
	[La(pym-mix)]^+^	0.89 ± 0.05	12.0 ± 0.05	10.7
	[La(pazi)]^+^	0.91 ± 0.03	11.9 ± 0.05	10.5
Ba^2+^	[Ba(macropa)]	1.17 ± 0.03	11.0 ± 0.10	8.2
	[Ba(pym-2,4)]	0.99 ± 0.04	10.1 ± 0.11	8.8
	[Ba(pym-2,6)]	0.85 ± 0.03	10.2 ± 0.08	8.8
	[Ba(pym-mix)]	1.09 ± 0.04	10.3 ± 0.16	8.9
	[Ba(pazi)]	1.09 ± 0.09	10.1 ± 0.27	8.7

Among the individual chelators, the range of the stability constants is remarkably narrow for a given metal ion. By contrast, a significant disparity is apparent between the different metal ions. That is to say, complex stability constants increase in the order Ba < La < Pb. All complex stability constants determined with the new ligands are lower compared to corresponding metal complexes of macropa, caused by the higher p*K*_a_ values of macropa.

For a more realistic comparison of the complex formation at certain pH values, the pH-dependent binding affinities *K*_d,M_ of the metals (M) were determined (last column of Table 2) by eqs 1–3:

1 $$K_{d,M}=\frac{1+10^{pK_{a,1}-pH}+10^{pK_{a,2}+pK_{a,1}-2pH}}{K_{M}}$$

2 $$K_{d,M}=\frac{1+10^{pK_{a,4}-pH}+10^{pK_{a,3}+pK_{a,4}-2pH}+10^{pK_{a,2}+pK_{a,3}+pK_{a,4}-3pH}}{10^{\log K}}$$

3 $$pK_{d,M}=-log_{10}\left(K_{d,M}\right)$$

where the pH acts as the variable (*x* value), *K*_M_ is the respective decadic exponent of the complex stability constant log *K* of the metal (M), and the p*K*_a,*i*_ (*i* = 1–4) values are the chelator’s respective protonation constants extrapolated to zero ionic strength. For better comparability, *K*_d,M_ is transformed to p*K*_d,M_. The p*K*_d,M_ values were calculated for the pH range 4–14. Exemplarily for Ba^2+^, the pH-dependent binding affinities for the novel chelators **pym-2,4** (blue), **pym-2,6** (orange), **pym-mix** (green), and **pazi** (magenta), as well as macropa (gray) and DOTA (black) are shown in Figure 4.

**Figure 4:**
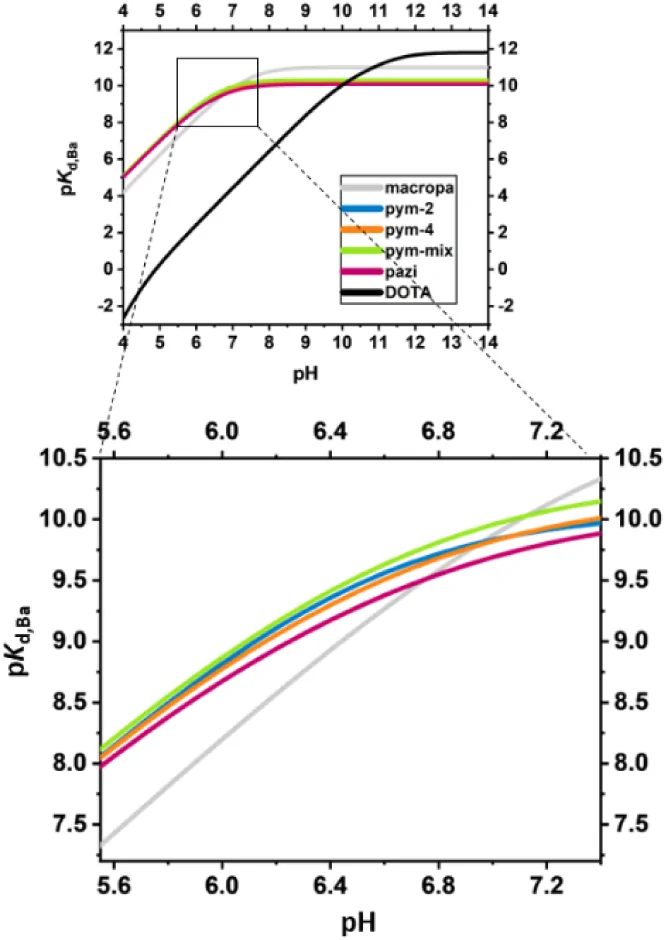
Binding affinity p*K*_d,Ba_ for Ba^2+^, calculated for the pH range 4–14, for macropa (gray), **pym-2,4** (blue), **pym-2,6** (orange), **pym-mix** (green), **pazi** (magenta), and DOTA (black). The pH range 5.5–7.4 is magnified and given as augmentation.

Upon increasing pH, all chelators show a steady increase in their binding affinity as they successively deprotonate according to their individual p*K*_a_ values, with concomitantly decreasing the competition between H^+^ and the metal ions for binding to the chelators’ functional groups. Determined by the highest p*K*_a_, the (pH-dependent) binding affinity p*K*_d,M_ asymptotically approaches the (pH-independent) stability constant log *K*. A closer focus was placed especially on the pH range between 5.5 and 7.4, as it is examined for typical radiolabeling conditions and biological systems.

In this important pH range, the p*K*_d,Ba_ values reveal that Ba^2+^ binds to macropa with an affinity several orders of magnitude higher than to DOTA. This agrees well with the poor dissolution of BaSO_4_ by DOTA in contrast to macropa at pH 6, even though DOTA has the highest stability constant reported for barium (log *K* = 11.8).^49,55^ Therefore, the pH-dependent binding affinity p*K*_d,Ba_ is the more appropriate quantity to assess complexation properties for radiopharmaceutical applications at typical radiolabeling conditions than the stability constant log *K*. The obtained better radiolabeling results shown below for the novel chelators with ^131^Ba compared to macropa are in perfect agreement. The following trend for affinity to Ba^2+^ at pH 6 is found to be **DOTA < macropa < pym-(2,4/2,6/mix)**.

Furthermore, up to pH = 6.7, all four novel chelators show a higher binding affinity to Ba^2+^ than macropa even though their log *K* values are generally lower, which originates from their lower p*K*_a_ values. Correspondingly, macropa’s affinity toward Ba^2+^ exceeds that of **pazi** at pH > 6.7, that of **pym-2,4** and **pym-2,6** at pH > 6.9, and that of **pym-mix** at pH > 7.1.

### Radiolabeling and Complex Stability

Radiolabeling was carried out to demonstrate the applicability of the novel chelators for radiopharmaceutical purposes with different radionuclides. Therefore, ^203^Pb, ^212^Pb, ^133^La, ^225^Ac, ^131^Ba, and ^223^Ra were incubated with different concentrations (1·10^–3^–5·10^–8^ M) of the respective chelator at 25 °C. Two recently developed TLC systems (NP-TLC and RP-TLC) were used to monitor the progress of radiolabeling.^32,39^ Additionally, macropa was labeled with all radiometals both to validate already reported results^15,18,22,32,39^ and to establish an internal standard for quality control of the radiometals.

A quantitative radiolabeling with ^133^La and ^225^Ac was observed for all chelators down to 10^–6^ M chelator concentration (Figure 5, SI: Table S9), which, however, declined at 10^–7^ M. ^133^La was able to still label quite well at this concentration, so that similar results were obtained with **pym-mix** (66% RCC) compared to macropa (64% RCC), but with significant improvement using **pym-2,4** (97% RCC) and **pym-2,6** (86% RCC), however low RCC was observed with **pazi** (37%). For ^225^Ac, there was almost no complexation at 10^–7^ M. It is imperative to note that the radiolabeling outcomes obtained for ^133^La and ^225^Ac are divergent, although the two elements are discussed as being chemically identical.^20–22^ The influence of the different physical half-lives, 3.92 h for ^133^La and 10.9 d for ^225^Ac, has to be considered, because the activity of a radionuclide is proportional to the amount of substance (A = λ·N) and is in direct correlation with the half-life (λ = ln2/*T*_1/2_).^56^ Thus, the amount of substance of a radionuclide with a shorter half-life is generally lower at the same activity. In the case of ^225^Ac and ^133^La, there is a difference of 2 orders of magnitude in the amount of substance at the same activity amount.

**Figure 5:**
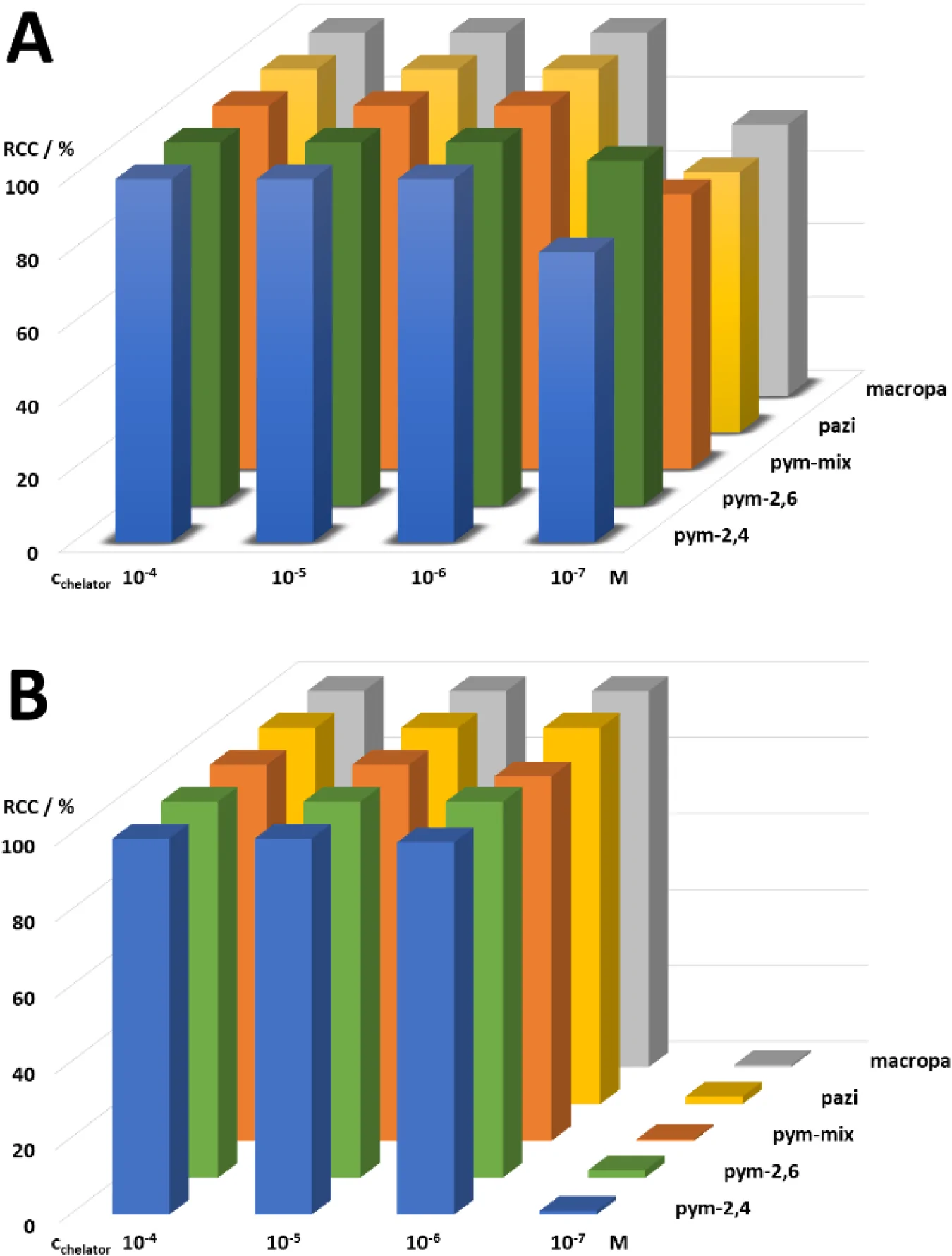
Radiolabeling of **pym-2,4** (blue), **pym-2,6** (green), **pym-mix** (orange), and **pazi** (yellow) compared to macropa (gray) with 100 kBq of ^133^La (A) and ^225^Ac (B) in a serial dilution from 10^–4^ to 10^–7^ M in 100 μL total volume. RCC’s were determined after 1 h reaction time at 25 °C using the NP-TLC system.

Remarkable radiolabeling results at chelator concentrations down to 10^–7^ M were obtained for both Pb isotopes ^212^Pb (*T*_1/2_ = 10.6 h) and ^203^Pb (*T*_1/2_ = 51.9 h) under mild conditions (Figure 6, SI: Table S10), but the same phenomenon of different amounts of substance at the same activity amount occurs. In this case, the half-lives and nuclear properties are different, but the chemical properties are identical. A slightly better radiolabeling with ^212^Pb was observed compared to ^203^Pb. It is noteworthy that the triplicate ^203^Pb labeling shown here was carried out 2–3 half-lives after radionuclide production. Of note, a better radiolabeling performance can be obtained when the radiolabeling was done with freshly prepared ^203^Pb.

**Figure 6:**
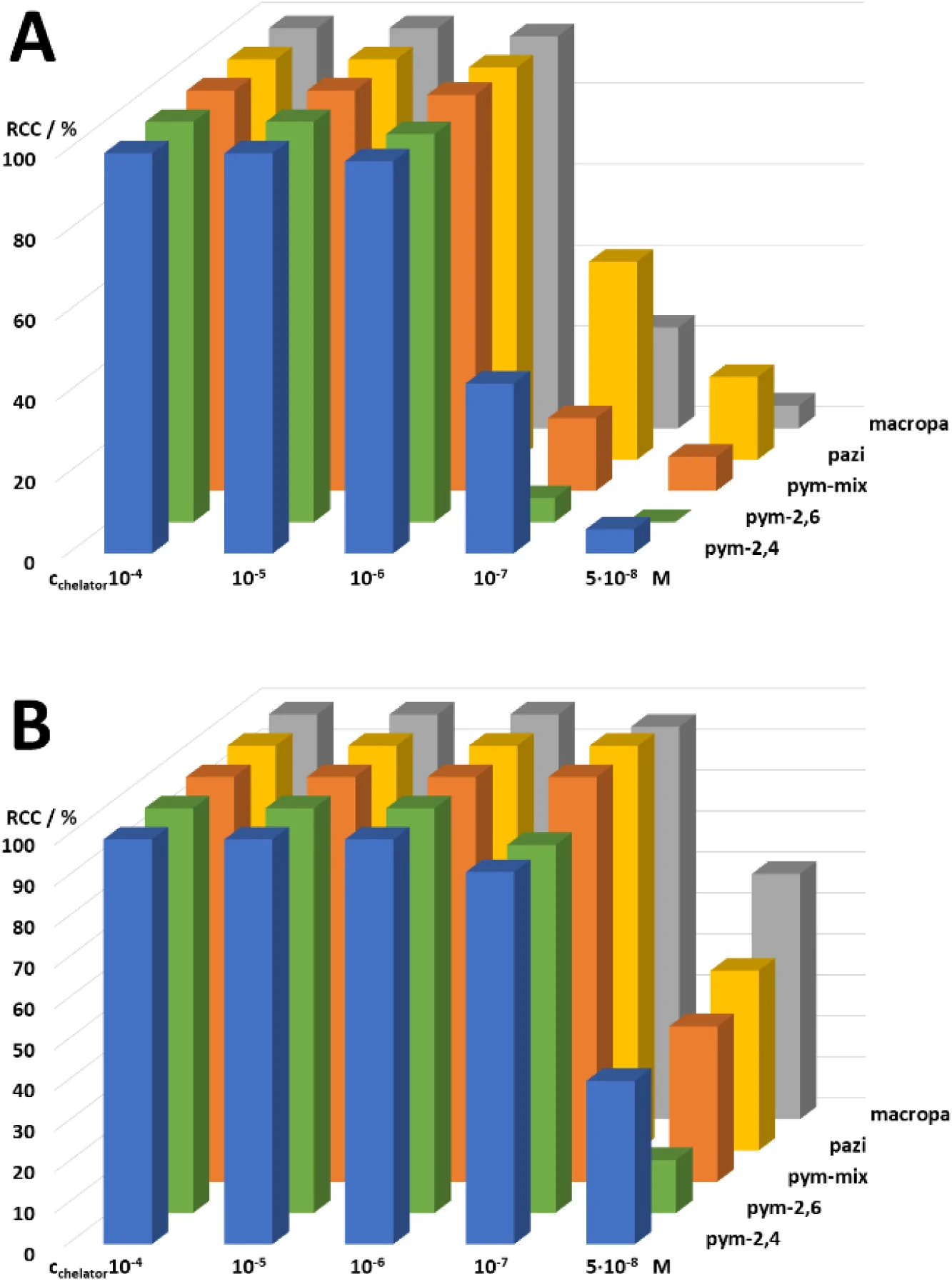
Radiolabeling of **pym-2,4** (blue), **pym-2,6** (green), **pym-mix** (orange), and **pazi** (yellow) compared to macropa (gray) with 100 kBq of ^203^Pb (A) and ^212^Pb (B) in a serial dilution from 10^–4^ to 5·10^–8^ M. RCCs were determined after 1 h reaction time at 25 °C using the NP-TLC system.

Exceptional outcomes were accomplished with the radiolabeling of ^131^Ba and ^223^Ra (Figure 7, SI: Table S11). For ^131^Ba, all four new chelators demonstrated a high radiochemical conversion of over 90% in both TLC systems at a 10^–6^ M concentration. In contrast, macropa exhibited a conversion of 75%, while challenging the complexes with a 50 mM EDTA solution (RP-TLC system) resulted in only 12% radiochemical conversion. Even down to 10^–7^ M, over 75% radiochemical conversion was obtained for all the novel chelators, following the trend: **pym-mix** < **pazi** < **pym-2,6** < **pym-2,4**. For **pym-2,4**, an astonishing mean radiochemical conversion of over 90% was found for ^131^Ba at 10^–7^ M, which is ideal for future *in vivo* applications.

**Figure 7:**
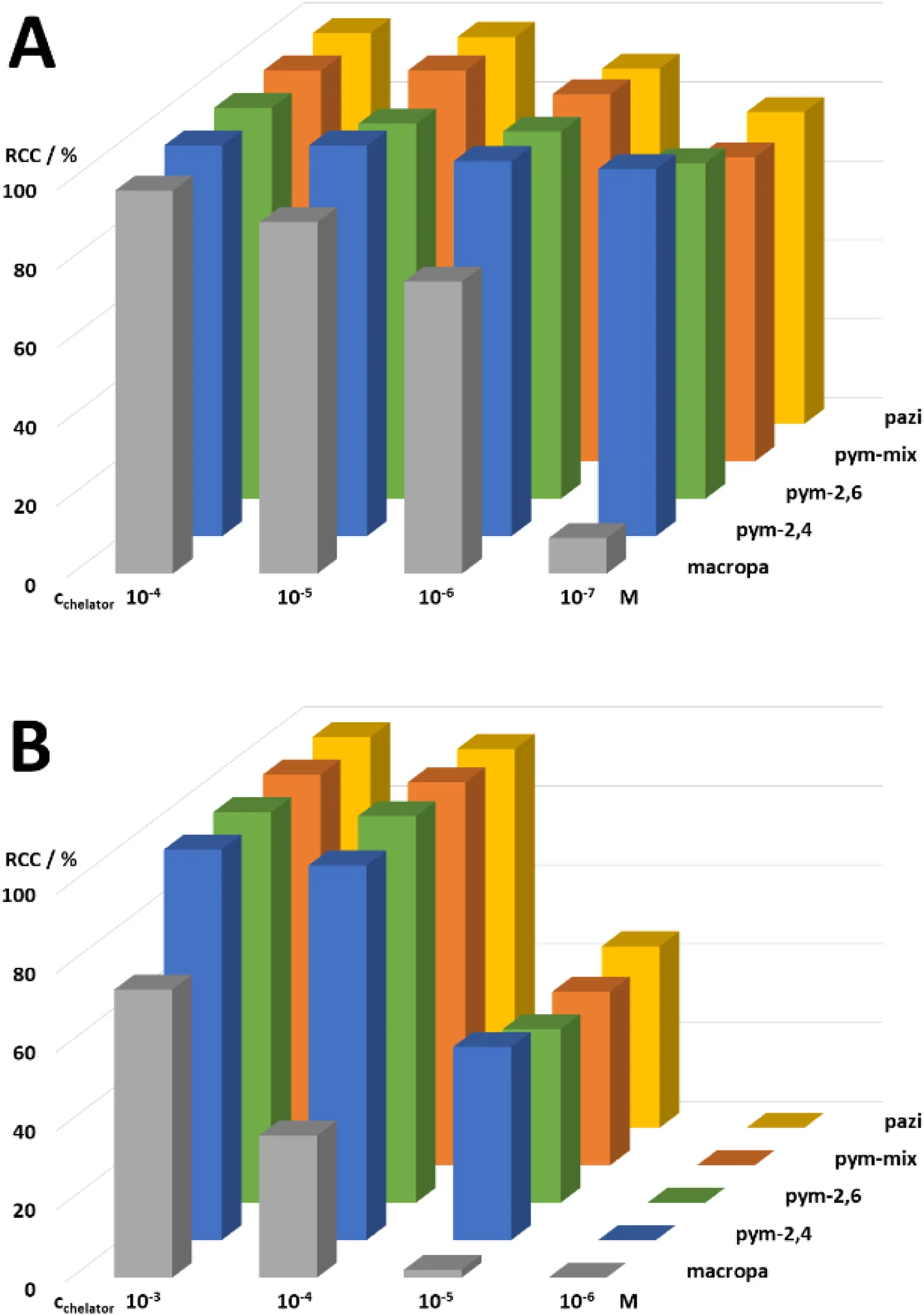
Radiolabeling of **pym-2,4** (blue), **pym-2,6** (green), **pym-mix** (orange), and **pazi** (yellow) compared to macropa (gray) in a serial dilution from 10^–3^ to 10^–7^ M chelator concentration with 100 kBq of [^131^Ba]­Ba^2+^ (A) and with 10 kBq of [^223^Ra]­Ra^2+^ (B) in a total volume of 100 μL. RCCs were determined after 1 h reaction time at 25 °C (NP-TLC system).

Furthermore, the ^223^Ra-labeled complexes were diluted in PBS and human serum and stirred at 37 °C up to 14 days (Figure 8 and SI: Table S12). The stability of the respective ^223^Ra-labeled complexes was investigated by the detection of the fraction of intact radiotracer using the NP-TLC system. In the case of [^223^Ra]­[Ra­(macropa)], a complete dissociation was observed after 8 days. In contrast, all the ^223^Ra-labeled complexes prepared with the new chelators showed an extraordinary stability in human serum (>80% for **pym-mix** and >90% for all other chelators) over 14 days.

**Figure 8:**
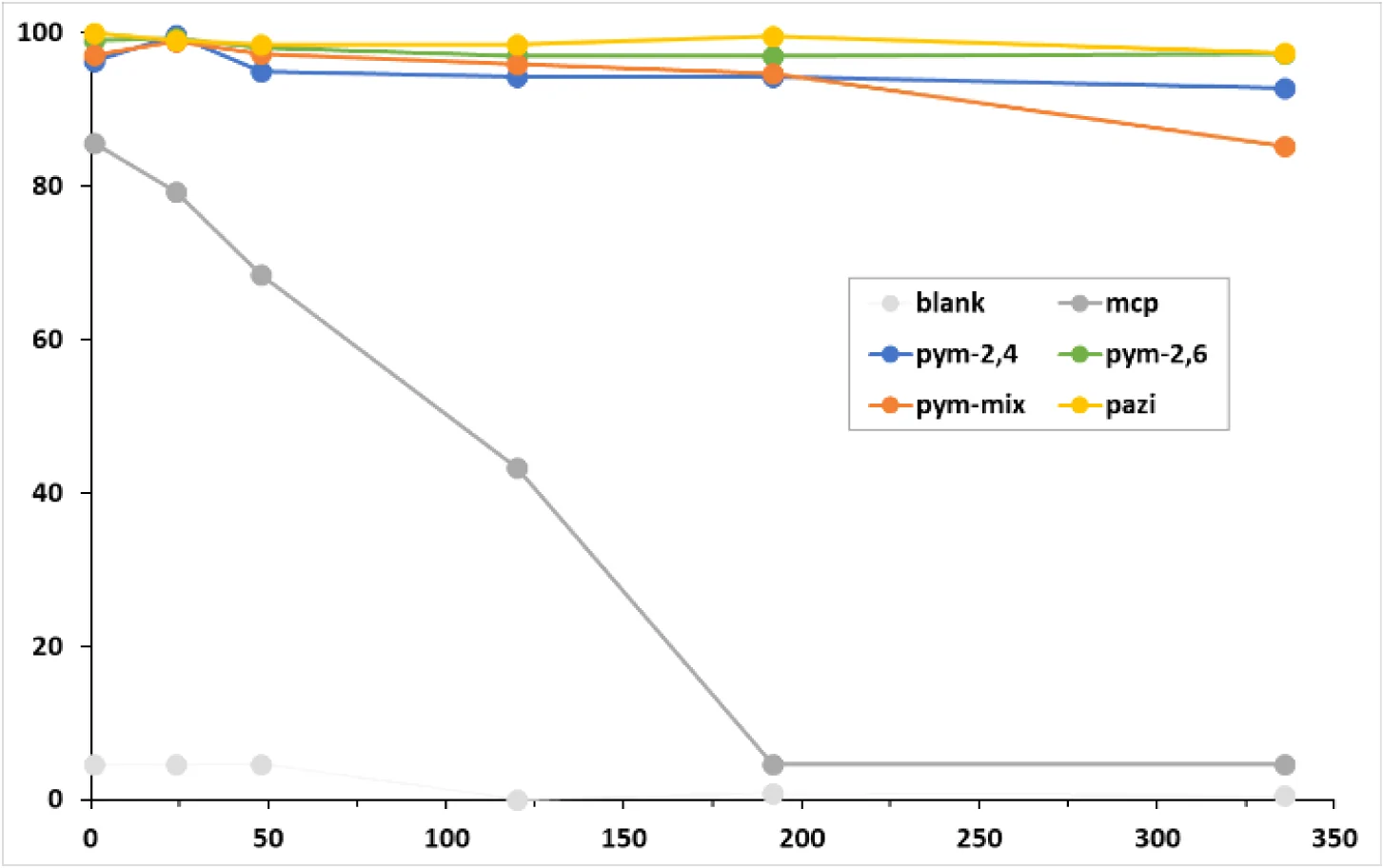
Stability test of the ^223^Ra-labeled complexes (10^–3^ M, 5 kBq, 50 μL) diluted in PBS (150 μL) and human serum (250 μL) and stirred at 37 °C for 14 d (336 h). The fraction of the stable ^223^Ra complexes was determined via NP-TLC.

### Structural Investigations of the Complexes

Upon investigating the structural characteristics of the complexes forming with the novel chelators including macropa as reference system, we aimed to figure out the potential origins that could explain observed peculiarities in the complexes’ kinetic and thermodynamic behavior. Therefore, complexes with Pb^2+^, Ba^2+^, Ra^2+^, La^3+^, and Ac^3+^ were optimized by DFT calculations. The results are discussed in consideration of experimental NMR data collected for the complexes obtained with Pb^2+^, La^3+^, and Ba^2+^.

At given pD 6.0, each chelator shows two signals in the aromatic region (δ_H_ 7.5–9.0 ppm) except of **pym-mix**, which shows four (Figure 9). In the aliphatic region, the macrocycle gives rise to three ^1^H signals in the range δ_H_ 4.0–3.3 ppm. One singlet for the methylene protons of the OCH_2_CH_2_O moiety and one broadened triplet for each methylene group of the OCH_2_CH_2_N moiety. The small number of signals due to the macrocycle indicates a highly dynamic behavior in solution with rapid interconversion and thus averaging over a large conformational space. Additionally, the signal of the benzylic methylene hydrogens appears as one singlet, in acidic solution at δ_H_ 4.6–4.9 ppm close to the HDO resonance, and thus obscured or truncated from solvent signal suppression. Upon increasing pD, the signal not only shows pD-dependent shift to higher field, but also severe broadening (cf. Figure S1, SI).

**Figure 9:**
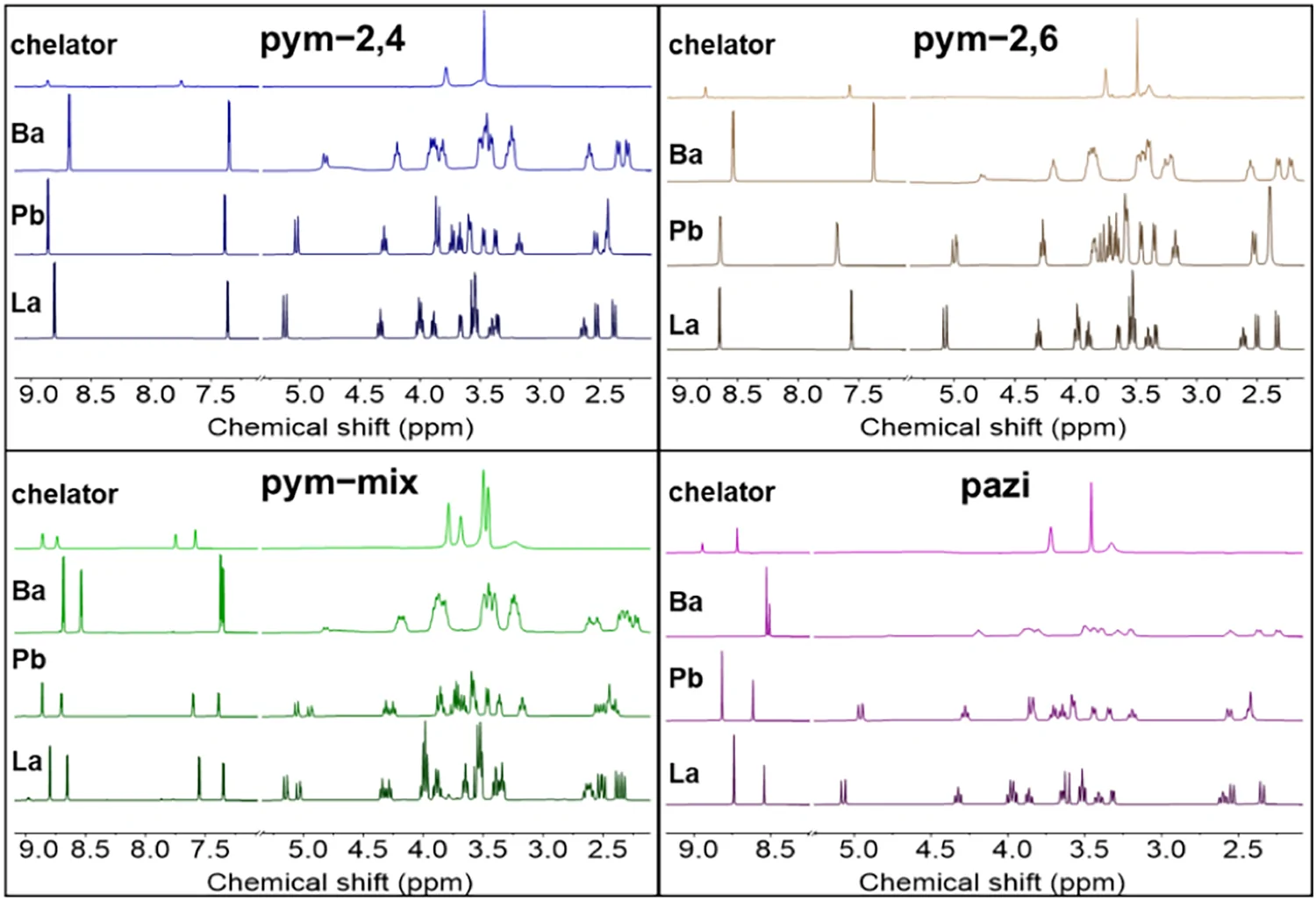
^1^H NMR spectra of the complexes formed with the new chelators: **pym-2,4** (blue, top left), **pym-2,6** (brown, top right), **pym-mix** (green, bottom left), and **pazi** (magenta, bottom right) obtained from 12.5 mM chelator, from top to bottom, in absence of metal as well as in the presence of 15.0 mM (1.2 equiv) Ba^2+^, Pb^2+^, and La^3+^, respectively, in D_2_O solution at pD 6.0 at (25 ± 1) °C. For clarity, the spectral regions from 2.0 to 5.5 ppm and from 7.0 to 9.2 ppm are shown. The HDO residual signal was removed by signal suppression.

Upon complexation of metal ions at pH 6—involving potential decadentate coordination by the chelator—the conformational variability is drastically reduced as the complex structure becomes more rigid. Consequently, the hydrogen atoms of all methylene groups become diastereotopic, accompanied by considerable signal displacements. The thus resulting 12 proton resonances of the macrocycle along with the two doublets associated with the diastereotopic benzylic methylene hydrogens, but only one single set of signals for the aromatic rings indicating equivalence, evidences the complexes to be of effective *C*_2_ symmetry. The asymmetry arising from the two distinct heteroaromatic residues in **pym-mix** causes virtually a doubling of the number of all signals, comparable to a superposition of spectra of the two parenting, symmetrically substituted chelators. Corresponding ^13^C NMR data along with assignment of all ^1^H, ^13^C, and ^15^N NMR chemical shifts for the complexes of Ba^2+^, Pb^2+^, and La^3+^ with all novel chelators are summarized in the SI.

In order to get a deeper insight into the bonding characteristics of the formed complexes, ^15^N NMR chemical shifts of the free chelators as well as of the corresponding Ba-, Pb-, and La-complexes were determined, which are summarized in Tables S4–S6, SI. The resonance of the aromatic nitrogen atom in position 2 reveals the largest shifts Δδ (between 5 and 17 ppm) for complexes of La^3+^, while for complexes of Ba^2+^ and Pb^2+^ it is 6 and 7 ppm at most, compared to the corresponding free chelators at pH = 4. For the macrocyclic amine nitrogen atom, minimal differences in the ^15^N chemical shifts occurred compared to those of the free chelators. This is attributed to similar inductive effects the amine nitrogen experienced when bound to either a proton at pH 4 (for the free chelator), or to any of the metal ions explored here. However, δ_N_ values of 40 and 42 ppm observed in Ba-complexes of **pym-2,4** and macropa, respectively, are more characteristic for non-coordinating (deprotonated) amine nitrogen as reflected by macropa’s δ_N_ of 39 ppm at pH 11 vs. 48 ppm at pH 4; cf. Table S4, SI).

Finally, ^207^Pb NMR spectra were acquired (Figure S3, SI) to address complexation behavior from the metal ion’s perspective, taking advantage of the NMR-active spin-1/2 nucleus ^207^Pb.^57^ All complexes studied give rise to signals downfield shifted (at least 650 ppm) from that of the Pb^2+^ aqua ion; see Table 3.

**Table 3:** Determined ^207^Pb NMR Chemical Shifts of the Aqueous Pb-Complexes Formed with the Various Chelators^a^

Ligand	δ [ppm]
**(H**_**2**_ **O)**_**n**_	–2957
**macropa**	–1953
pym-2,4	–2263
pym-2,6	–2309
**pym-mix**	–2287
**pazi**	–2231

a The aqua complex serves as a secondary reference.

Compared to the [Pb­(macropa)]^0^(aq) complex, the presence of a second aromatic ring nitrogen atom in the novel complexes causes an upfield shift between 280 ppm (**[Pb­(pazi)]**) and 360 ppm (**[Pb­(pym-2,6)]**) depending on its individual position, which corresponds to inductive and mesomeric effects on electron density redistribution and delocalization. Interestingly, the observed ^207^Pb NMR chemical shift for the Pb^2+^ complex with **pym-mix** is the exact average of that observed for the complexes formed with the parental chelators **pym-2,4** and **pym-2,6**, demonstrating the additivity of the individual contributions from the chelating side arms. The same observation applies to ^15^N chemical shifts of all nitrogen atoms for the three pym-derivatives (cf. Tables S4–S6, SI).

Based on these results, one can say that the second ring nitrogen atom leads to a decrease in electron density of the aromatic ring and renders the donor atoms to be harder Lewis bases, yet the novel chelators cause a shielding of the Pb nucleus, which appears to be somehow destabilizing (cf. Table 2). Apparently, the electron density redistribution is more complex and requires additional bond analyses which are beyond the scope of the present work. At this point we speculate whether increasing the electron density in the aromatic ring thus further adds to the complex stability and better adapts the HSAB borderline character of Pb.

DFT calculations allowed valuable insights into the structural characteristics of the complexes, and are in very good agreement with calculations reported for macropa complexes.^32,58^ These analyses demonstrate that different metal ions exhibit a distinct coordination behavior. Nevertheless, the complexes of the new chelators presented here retain the coordination type observed in crystal structures determined for [M­(macropa)]^n+^ complexes^49,58,59^ and appear to be similar to the [M­(macropa)]^n+^ complexes reported previously.^32^ The metal ions’ position slightly above the plane of the macrocyclic ring system is surrounded by heteroaromatic diazine carboxylate residues from above the plane. Figure 10 exemplarily shows the DFT-calculated structures of metal complexes forming with **pym-2,4**, while those of the other novel chelators and macropa combined with the metal ions of interest are depicted in Figure S27, SI.

**Figure 10:**
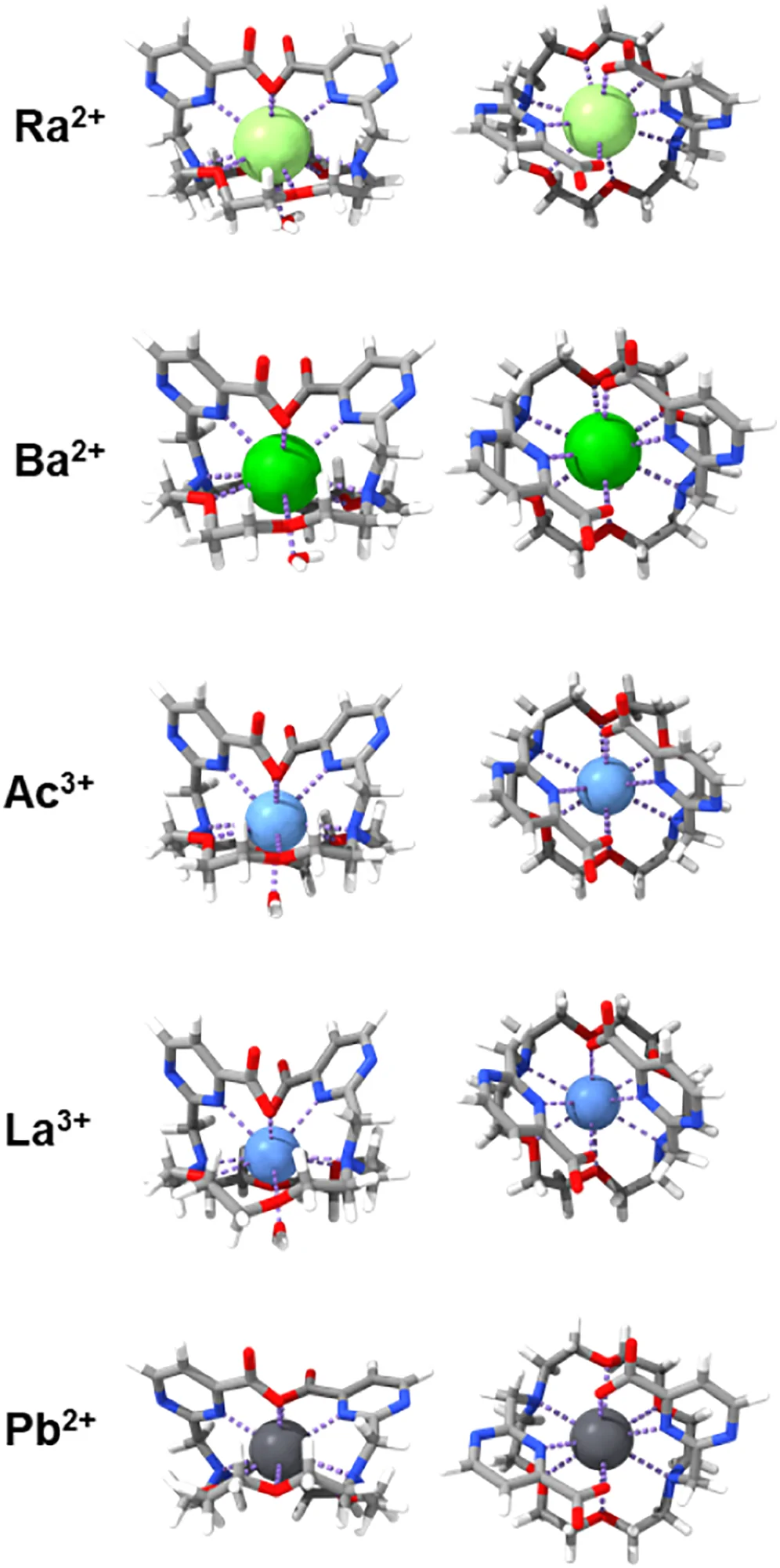
Side and top views of geometry-optimized [M­(pym-2,4)­(H_2_O)]^(*m*−2)+^ complexes (M = Ra^2+^, Ba^2+^, La^3+^, and Ac^3+^) and **[Pb­(pym-2,4)]** in aqueous solutions.

The initial coordination sphere incorporates one water molecule. However, the water molecule in the Pb-complexes exhibits a divergent behavior. Instead of coordinating to the metal center through its oxygen atom, it forms a hydrogen bond to two oxygen atoms of the chelators. As a result, a total coordination number of 8 was found with one amine nitrogen atom (N_amine_) and one ether oxygen atom of the macrocycle (O_macro_) that are not coordinating in [Pb­(macropa)].^32^ However, NMR experiments (Figure 9) indicate an overall *C*_2_ symmetry as inferred from the number of signals. Therefore, DFT calculations for Pb-complexes were repeated without the coordinating water molecule to reduce the influence of potentially induced hydrogen bonding between the water molecule and the macrocyclic ether oxygen atoms. The thus obtained results now suggest that in fact a higher symmetry can be achieved, indicating a total coordination number of 10 for [Pb­(macropa)] and also for all four Pb-complexes formed with the new chelators (Figure 10 and Figure S27, SI).

The bond lengths of the coordinating nitrogen atoms in the aromatic ring to the metal ion (M–N_arom_) display a satisfactory correlation with the radii of the metal ions (cf. Figure 11 as well as Figures S23–S26, SI). In contrast, the bond lengths of the metal ion and the oxygen atom of the carboxylate (M–O_carbox_) reveal a significant variation between the metals. Nevertheless, the bond to the negatively charged carboxylate donor is the shortest for all metal ions, meaning that the interaction is the strongest and likely of ionic nature, underscoring the importance of negatively charged donor atoms in stabilizing these specific metal ions.^37^ Interestingly, the calculations point out that the individual metal ions are positioned not fully symmetrically with respect to the macrocycle, causing distinct bond lengths for corresponding donor sites as seen for the amino nitrogen atoms (M–N_amine_) and ether oxygen atoms (M–O_macro_), respectively. Correspondingly, those complexes showing the smallest variance in these bond lengths, leading to the closest convergence to *C*_2_ symmetry, also exhibit sharp ^1^H NMR signals (cf. Figure 9). High symmetry is especially found in the calculated La- and Ac-complexes and corroborated by NMR spectra of the La-complexes.

**Figure 11:**
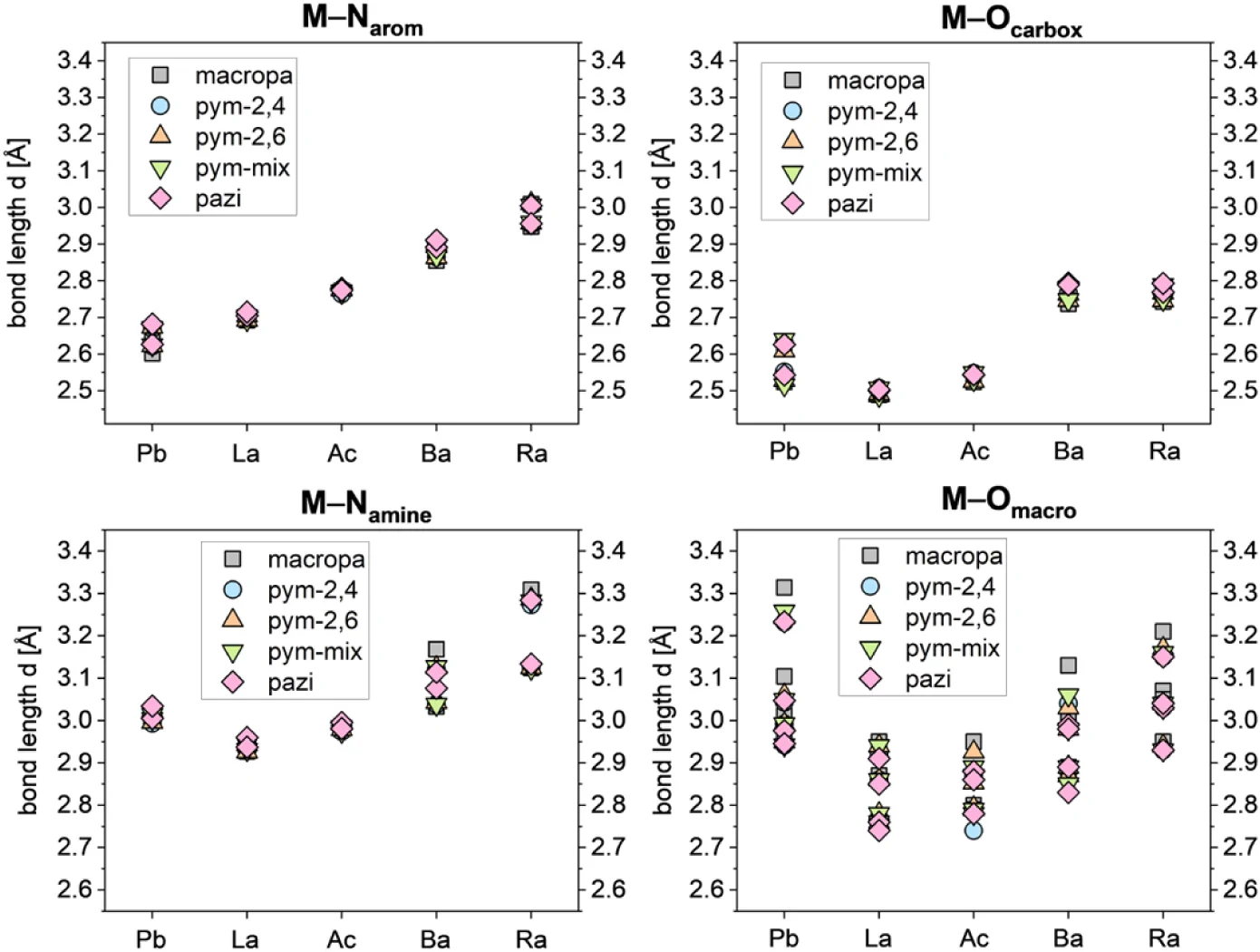
Bond lengths d in Å of the donor atoms (N_arom_: aromatic nitrogen atoms, N_amine_: amine nitrogen atoms, O_carbox_: carboxylic oxygen atoms, and O_macro_: ether oxygen atoms) of each chelator: **pym-2,4** (blue), **pym-2,6** (orange), **pym-mix** (green), and **pazi** (magenta) in comparison to macropa (gray) forming a complex with the metal ions Pb^2+^, La^3+^, Ac^3+^, Ba^2+^, and Ra^2+^ (sorted by increasing ionic radii^60^).

In contrast, the spectra of the Ba-complexes are characterized by broad signals, particularly those associated with the macrocyclic ring, in line with the determined asymmetry of the Ba-complexes. For the Pb-complexes, the obtained signals of the NMR spectra are sharper than for the Ba-complexes, but broader than for the La-complexes, which correlates with the calculated bond lengths showing a slight variance and also longer bond lengths than for the La-complexes especially for M–O_carbox_. This can be explained by the borderline character of this metal ion according to the HSAB principle, preferring soft donors (like nitrogen or even sulfur) instead of hard donors (like oxygen).^61^ Furthermore, the average coordination number of Pb^2+^ is 6, while the chelators discussed here are decadentate, leading to a plethora of donors for the metal ion and possibly to more intramolecular dynamics. These observations prompt the hypothesis that intramolecular dynamic processes in solution average out the metal ion position within the chelate cavity, thus giving rise to spectra that reflect symmetric structures.

## Conclusion

The present study demonstrates that systematic modification of the macropa scaffold through incorporation of diazine-based donor arms which leads to the four novel macrocyclic complexing agents macropyma-2,4 (**pym-2,4**), macropyma-2,6 (**pym-2,6**), macropyma-mix (**pym-mix**), and macropazi (**pazi**) provides a powerful strategy for tuning and improving the coordination chemistry of large radiometal ions. Although the additional aromatic nitrogen atom generally decreases ligand basicity and lowers thermodynamic stability constant relative to macropa, the resulting new diazine-based chelators exhibit superior pH-dependent binding affinities toward Ba^2+^ and excellent radiolabeling performance with ^131^Ba and ^223^Ra in addition to the still high binding affinities toward La^3+^ and Pb^2+^ combined with a high radiolabeling performance of ^133^La, ^225^Ac, and ^203/212^Pb. Most notably, the new ligands form highly inert ^223^Ra complexes that remain largely intact in human serum for at least 14 days, representing a significant advance in the development of molecular chelators for radium.

Beyond the radiochemical results, the combined thermodynamic, spectroscopic, and computational investigations provide valuable insight into the structural factors governing complex stability. The data suggest that efficient stabilization of large alkaline-earth and actinide ions requires not only strong metal–donor interactions but also an appropriate balance between ligand basicity, donor hardness, and geometric adaptation of the macrocyclic cavity. Furthermore, the observed differences between Ba^2+^ and Ra^2+^ complexation behavior indicate that barium should be regarded as a useful, but not necessarily perfect, nonradioactive surrogate and diagnostic counterpart for radium. These findings establish design principles for the future development of chelators for targeted alpha therapy and identify diazine-functionalized macropa analogues as promising platforms for theranostic applications involving ^223^Ra/^224^Ra and ^131^Ba.

## Experimental Section

### General Information

All chemicals used for the syntheses were purchased from commercial suppliers and used without further purification. NMR samples were prepared using deuterated solvents (deutero GmbH) D_2_O (99.95% D) and D_2_O solutions of both DCl (38% in D_2_O with 99% D) and NaOD (40% in D_2_O with 99% D). pD values were measured using a pH meter (VWR pHenomenal MU 6100 L) with a pH electrode (WTW SenTix Mic) and corrected using the formula: *pD = pH + 0.44*.^62^ A 1 mM stock solution of each chelator with 0.1 M NaCl was prepared in D_2_O, with pD adjusted between 0.7 and 11.3 in 1 mL volumes. High-purity metals (99.99%) were weighed with plastic spatulas. For NMR experiments, metal salt stock solutions (La^3+^, Pb^2+^, Ba^2+^) with a concentration of 100 mM were prepared in D_2_O. Samples used for ligand characterization were prepared with 10 mM of ligand at pD 4. Samples aiming at structure elucidation of metal chelates were prepared with a ligand concentration of 12.5 mM and a metal ion concentration of 15 mM at pD 6. For ITC experiments, metal salts were dissolved in Milli-Q water (Merck Millipore). ITC measurements were performed at 25 °C with 0.1 M NaCl ionic strength.

The synthesis of macropa^32,39^ as well as the synthesis of the aromatic side-arms **2a** and **2b** ^52^ is reported elsewhere. The general synthesis steps are shown in Figure 2, and the corresponding analytical data are given in the SI.

**Methyl 6-(bromomethyl)­pyrazine-2-carboxylate (2c).** Methyl 6-methylpyrazine-2-carboxylate (1.23 g, 8.08 mmol) and *N*-bromosuccinimide (1.29 g, 7.28 mmol) were dissolved in CCl_4_ (10 mL) and the solution was then heated to 80 °C. Afterward, azobis­(isobutyronitrile) (398 mg, 2.43 mmol) was added and the reaction was stirred at 80 °C for 16 h. After cooling to rt, the solvent was removed and the crude product was purified by automated column chromatography (silica gel, 0/100 → 40/100 petroleum ether/ethyl acetate). Compound **2c** was obtained in 19% (351 mg) yield as a yellow oil. Additionally, 529 mg of the starting material (conversion: 57%) were recovered and the double-brominated by-product (320 mg, 13%) was obtained as well. ^1^H NMR (400 MHz, CDCl_3_): δ = 9.17 (d, ^4^ *J* = 0.6 Hz, 1H, H_Ar_), 8.89 (d, ^4^ *J* = 0.6 Hz, 1H, H_Ar_), 4.60 (s, 2H, CH_2_Ar), 4.00 (s, 3H, CH_3_) ppm. ^13^C NMR (101 MHz, CDCl_3_): δ = 164.0 (C=O), 152.5 (C_Ar_), 148.0 (CH_Ar_), 144.9 (CH_Ar_), 142.2 (C_Ar_), 53.3 (CH_3_) 29.5 (CH_2_Ar) ppm. MS (ESI+): *m/z* = 231 [M + H]^+^ (^79^Br), 233 [M + H]^+^ (^81^Br).

### General Synthesis of the Macrocycle with the Side Arms

Kryptofix 2.2 (**1**) (1 equiv), *N*,*N*-diisopropylethylamine (DIPEA, 3 equiv) and the respective methyl 6-(bromomethyl)­(diazine)-carboxcylates **2a**–**c** (2.1 equiv) were dissolved in anhydrous acetonitrile and the mixture was stirred at 75 °C for 16 h. After cooling to rt, the solvent was removed and the crude product was purified by automated column chromatography (silica gel, 0/100 → 20/100 ethyl acetate +1% NEt_3_ / ethanol +1% NEt_3_). Compounds **3a**–**c** were obtained as yellow oils.

**Dimethyl 2,2′-((1,4,10,13-tetraoxa-7,16-diazacyclooctadecane-7,16-diyl)­bis­(methylene))-bis­(pyrimidine-4-carboxylate) (3a).** Methyl 2-(bromomethyl)­pyrimidine-4-carboxylate (**2a**, 380 mg, 1.65 mmol), Kryptofix 2.2 (**1**, 173 mg, 0.66 mmol) and DIPEA (345 μL, 1.98 mmol) were reacted in 15 mL of acetonitrile to obtain **3a** in a yield of 250 mg (67%). ^1^H NMR (400 MHz, CDCl_3_): δ = 8.94 (dd, ^3^ *J* = 5.0 Hz, ^4^ *J* = 1.1 Hz, 2H, H_Ar_), 7.81 (d, ^3^ *J* = 4.9 Hz, 2H, H_Ar_), 4.18 (s, 4H, CH_2_Ar), 3.99 (s, 6H, CH_3_), 3.67 (t, ^3^ *J* = 5.7, 8H, OCH_2_), 3.57 (s, 8H, OCH_2_), 3.02 (t, ^3^ *J* = 5.7 Hz, 8H, NCH_2_) ppm; ^13^C NMR (101 MHz, CDCl_3_): δ = 169.5 (C_Ar_), 164.8 (C=O), 159.2 (CH_Ar_), 154.8 (C_Ar_), 118.6 (CH_Ar_), 70.6 (OCH_2_), 69.7 (OCH_2_), 61.3 (CH_2_Ar), 53.8 (NCH_2_), 53.3 (CH_3_) ppm. MS (ESI+): *m/z* = 562 [M]^+^.

**Dimethyl 4,4′-((1,4,10,13-tetraoxa-7,16-diazacyclooctadecane-7,16-diyl)­bis­(methylene))-bis­(pyrimidine-2-carboxylate) (3b).** Methyl 6-(bromomethyl)­pyrazine-2-carboxylate (**2b**, 380 mg, 1.64 mmol), Kryptofix 2.2 (**1**, 205 mg, 0.78 mmol) and DIPEA (409 μL, 2.35 mmol) were reacted in 15 mL of acetonitrile to obtain **3b** in a yield of 351 mg (80%). ^1^H NMR (400 MHz, CDCl_3_): δ = 8.83 (d, ^3^ *J* = 5.1 Hz, 2H, CH_Ar_), 8.04 (d, ^3^ *J* = 5.1 Hz, 2H, CH_Ar_), 4.04 (s, 6H, CH_3_), 3.96 (s, 4H, CH_2_Ar), 3.56 (t, ^3^ *J* = 5.4 Hz, 8H, OCH_2_), 3.49 (s, 8H, OCH_2_), 2.88 (t, ^3^ *J* = 5.4 Hz, 8H, NCH_2_) ppm; ^13^C NMR (151 MHz, CDCl_3_): δ = 172.0 (C_Ar_), 164.2 (C=O), 158.1 (CH_Ar_), 155.8 (C_Ar_), 122.2 (CH_Ar_), 70.8 (OCH_2_), 69.7 (OCH_2_), 60.7 (CH_2_Ar), 55.4 (NCH_2_), 53.7 (CH_3_) ppm. MS (ESI+): *m/z* = 562 [M]^+^.

**Dimethyl 6,6′-((1,4,10,13-tetraoxa-7,16-diazacyclooctadecane-7,16-diyl)­bis­(methylene))-bis­(pyrazine-2-carboxylate) (3c).** Methyl 4-(bromomethyl)­pyrimidine-2-carboxylate (**2c**, 205 mg, 0.89 mmol), Kryptofix 2.2 (**1**, 93 mg, 0.35 mmol) and DIPEA (186 μL, 1.06 mmol) were reacted in 10 mL of acetonitrile to obtain **3c** in a yield of 185 mg (79%).^1^H NMR (400 MHz, CDCl_3_): δ = 9.14 (s, 2H, CH_Ar_), 9.12 (s, 2H, CH_Ar_), 4.02 (s, 4H, CH_2_Ar), 4.00 (s, 6H, CH_3_), 3.62 (t, ^3^ *J* = 5.6 Hz, 8H, OCH_2_), 3.56 (s, 8H, OCH_2_), 2.88 (t, ^3^ *J* = 5.6 Hz, 8H, NCH_2_) ppm; ^13^C NMR (101 MHz, CDCl_3_): δ = 164.7 (C=O), 156.3 (C_Ar_), 148.7 (CH_Ar_), 144.1 (CH_Ar_), 141.5 (C_Ar_), 70.7 (OCH_2_), 69.8 (OCH_2_), 59.6 (CH_2_Ar), 54.69 (NCH_2_), 53.02 (OCH_3_) ppm. MS (ESI+): *m/z* = 562 [M]^+^.

**Methyl 4-((16-((4-(methoxycarbonyl)­pyrimidin-2-yl)­methyl)-1,4,10,13-tetraoxa-7,16-diaza-cyclooctadecan-7-yl)­methyl) pyrimidine-2-carboxylate (3d).** Kryptofix 2.2 (1, 2 eq., 175 mg, 0.67 mmol) and DIPEA (3 eq., 174 μL, 1.00 mmol) were dissolved in 50 mL of anhydrous acetonitrile and the mixture was heated to 75 °C. Methyl 4-(bromomethyl)­pyrimidine-2-carboxylate **2b** (1 eq., 77 mg, 0.33 mmol) dissolved in 20 mL of anhydrous acetonitrile was added over 5 h using a syringe pump and the mixture was finally stirred for 16 h at 75 °C. After cooling to rt, the solvent was removed and the crude product was purified by automated column chromatography (silica gel, 0/100 → 20/100 ethyl acetate +1% NEt_3_ / ethanol +1% NEt_3_). The resulting methyl 4-((1,4,10,13-tetraoxa-7,16-diazacyclooctadecan-7-yl) methyl)­pyrimidine-2-carboxylate was obtained (89 mg, 65%) as yellow oil. ^1^H NMR (600 MHz, CDCl_3_): δ = 8.90 (d, ^3^ *J* = 5.1 Hz, 1H, CH_Ar_), 7.86 (d, ^3^ *J* = 5.1 Hz, 1H, CH_Ar_), 3.94 (s, 3H, CH_3_), 3.86 (s, 2H, CH_2_Ar), 3.82 (t, ^3^ *J* = 4.6 Hz, 4H, OCH_2_), 3.53–3.49 (m, 4H, OCH_2_), 3.46 (t, ^3^ *J* = 4.8 Hz, 4H, OCH_2_), 3.43–3.38 (m, 4H, OCH_2_), 3.14 (t, ^3^ *J* = 4.8 Hz, 4H, NCH_2_), 2.76 (t, ^3^ *J* = 4.8 Hz, 4H, NCH_2_) ppm; ^13^C NMR (151 MHz, CDCl_3_): δ = 171.0 (C_Ar_), 164.0 (C=O), 158.1 (CH_Ar_), 155.4 (C_Ar_), 122.3 (CH_Ar_), 69.8 (OCH_2_), 68.4 (OCH_2_), 65.9 (OCH_2_), 58.8 (CH_2_Ar), 56.1 (NCH_2_), 53.4 (CH_3_), 48.3 (NCH_2_) ppm. MS (ESI+): *m/z* = 413 [M + H]^+^ (calculated: 412). Secondly, this intermediate compound (1 eq., 89 mg, 0.22 mmol), DIPEA (3 eq., 113 μL, 0.65 mmol) and methyl 2-(bromomethyl) pyrimidine-4-carboxylate **2a** (63 mg, 0.27 mmol) were dissolved in 10 mL of anhydrous acetonitrile and stirred at 75 °C for 16 h. After cooling to rt, the solvent was removed and the crude product was purified by automated column chromatography (silica gel, 0/100 → 20/100 ethyl acetate +1% NEt_3_ / ethanol +1% NEt_3_) to obtain **3d** (64 mg, 53%) as yellow oil. ^1^H NMR (400 MHz, CDCl_3_): δ = 8.95 (d, ^3^ *J* = 5.0 Hz, 1H, H5), 8.84 (d, ^3^ *J* = 5.1 Hz, 1H, H6′), 8.02 (d, ^3^ *J* = 5.1 Hz, 1H, H4), 7.83 (d, ^3^ *J* = 5.0 Hz, 1H, H4), 4.20 (s, 2H, H10), 4.03 (s, 3H, H20), 4.00 (s, 3H, H20′), 3.96 (s, 2H, H10′), 3.68 (t, ^3^ *J* = 5.8 Hz, 4H, H18), 3.57 (p, ^3^ *J* = 5.5 Hz, 12H, H13+15+16), 3.05 (s, 4H, H19), 2.85 (t, ^3^ *J* = 5.4 Hz, 4H, H12) ppm. ^13^C NMR (101 MHz, CDCl_3_): δ = 172.1 (C7′), 169.4 (C7), 164.9 (C3), 164.3 (C3′), 159.4 (C5′), 158.1 (C6), 155.8 (C1), 155.0 (C1′), 122.2 (C5′), 118.8 (C4), 70.9 (C15), 70.7 (C16), 69.9 (C18), 69.7 (C13), 61.2 (C10), 60.9 (C10′), 55.2 (C12), 54.1 (C19), 53.6 (C20), 53.5 (C20′) ppm. MS (ESI+): *m/z* = 563 [M + H]^+^.

### General Saponification Procedure

The bis­(methylesters) **3a**–**d** (1 equiv) and lithium hydroxide (6 equiv) were dissolved in a 1:1 methanol/water solution and stirred at 50 °C overnight. After cooling to rt, the solvent was removed and the crude product was purified by semi-preparative HPLC (Agilent Zorbax SB C-18 5 μm 80 Å, 9.4 × 250 mm, flow rate: 6 mL/min, gradient: 5/95 → 40/60 acetonitrile/water +0.1% TFA in 40 min). All chelators were obtained as colorless solids.

***N,N′*** **-Bis­[(4-carboxy-2-pyrimidinyl)­methyl]-1,10-diaza-18-crown-6 (pym-2,4).** LiOH (77 mg, 3.2 mmol) and **3a** (300 mg, 0.53 mmol) were reacted in 10 mL of a 1:1 methanol/water solution to yield 80 mg (28%) of **pym-2,4** after HPLC-purification. ^1^H NMR (400 MHz, D_2_O): δ = 8.80 (d, ^3^ *J* = 5.2 Hz, 2H, CH_Ar_), 7.68 (d, ^3^ *J* = 5.2 Hz, 2H, CH_Ar_), 4.68 (s, 4H, CH_2_Ar), 3.79–3.70 (m, 8H, O–CH_2_), 3.54 (t, ^3^ *J* = 4.9 Hz, 8H, NCH_2_), 3.36 (s, 8H, OCH_2_) ppm; ^13^C NMR (101 MHz, D_2_O): δ = 170.1 (C_Ar_), 161.3 (C=O), 160.0 (C_Ar_), 159.7 (CH_Ar_), 119.3 (CH_Ar_), 69.5 (OCH_2_), 64.0 (OCH_2_), 58.5 (CH_2_Ar), 54.8 (NCH_2_) ppm; ^15^N NMR (61 MHz): δ = 285.2 (N′_Ar_), 279.0 (N_Ar_), 46.5 (N_amine_) ppm. HRMS (ESI+): *m/z* = 535.2509 [M + H]^+^ (calcd. 535.2511).

***N,N′*** **-Bis­[(2-carboxy-4-pyrimidinyl)­methyl]-1,10-diaza-18-crown-6 (pym-2,6).** Compound **3c** (190 mg, 0.34 mmol) and LiOH (49 mg, 2.05 mmol) were reacted in a 1:1 methanol/water solution to yield 104 mg (57%) of **pym-2,6** after purification. ^1^H NMR (400 MHz, D_2_O): δ = 8.77 (d, ^3^ *J* = 5.2 Hz, 2H, CH_Ar_), 7.53 (d, ^3^ *J* = 5.2 Hz, 2H, CH_Ar_), 4.67 (s, 4H, CH_2_Ar), 3.80 (t, ^3^ *J* = 4.5 Hz, 8H, OCH_2_), 3.55 (t, ^3^ *J* = 4.9 Hz, 8H, NCH_2_), 3.49 (s, 8H, OCH_2_) ppm; ^13^C NMR (101 MHz, D_2_O): δ = 169.8 (C=O), 161.0 (C_Ar_), 159.6 (C_Ar_), 158.6 (CH_Ar_), 121.2 (CH_Ar_), 69.7 (OCH_2_), 64.0 (OCH_2_), 57.1 (CH_2_Ar), 54.6 (NCH_2_) ppm; ^15^N NMR (61 MHz): δ = 281.6 (N′_Ar_), 278.4 (N_Ar_), 45.6 (N_amine_) ppm. HRMS (ESI+): *m/z* = 535.2510 [M + H]^+^ (calcd. 535.2511).

***N,N′*** **-Bis­[(2-carboxy-6-pyrazinyl)­methyl]-1,10-diaza-18-crown-6 (pazi).** LiOH (12 mg, 0.5 mmol) and **3b** (35 mg, 0.062 mmol) were reacted in 5 mL of a 1:1 methanol/water solution to yield 31 mg (92%) of **pazi** after HPLC-purification. ^1^H NMR (400 MHz, D_2_O): δ = 8.99 (s, 2H, CH_Ar_), 8.69 (s, 2H, CH_Ar_), 4.69 (s, 4H, CH_2_Ar), 3.82–3.77 (m, 8H, OCH_2_), 3.55 (t, ^3^ *J* = 4.9 Hz, 8H, NCH_2_), 3.49 (s, 8H, OCH_2_) ppm; ^13^C NMR (101 MHz, D_2_O): δ = 169.8 (C=O), 149.5 (CH_Ar_), 148.2 (C_Ar_), 147.8 (CH_Ar_), 147.3 (C_Ar_), 72.3 (OCH_2_), 66.4 (OCH_2_), 58.0 (CH_2_Ar), 56.9 (NCH_2_) ppm; ^15^N NMR (61 MHz): δ = 325.1 (N′_Ar_), 323.8 (N_Ar_), 47.1 (N_amine_) ppm. HRMS (ESI+): *m/z* = 535.2511 [M + H]^+^ (calcd. 535.2511).

***N*** **-[(2-carboxy-4-pyrimidinyl)­methyl]-** *N′* **-[(4-carboxy-2-pyrimidinyl)­methyl]-1,10-diaza-18-crown-6 (pym-mix).** Compound **3d** (52 mg, 0.09 mmol) and LiOH (14 mg, 0.55 mmol) were reacted in 4 mL of a 1:1 methanol/water solution to yield 32 mg (65%) of **pym-mix** after HPLC-purification. ^1^H NMR (400 MHz, D_2_O): δ = 9.11 (d, ^3^ *J* = 5.1 Hz, 1H, CH_Ar_), 9.02 (d, ^3^ *J* = 5.2 Hz, 1H, CH_Ar_), 8.07 (d, ^3^ *J* = 5.1 Hz, 1H, CH_Ar_), 7.83 (d, ^3^ *J* = 5.2 Hz, 1H, CH_Ar_), 4.92 (s, 2H, CH_2_Ar), 4.89 (s, 2H, CH_2_Ar), 4.00–3.93 (m, 8H, OCH_2_), 3.76 (t, ^3^ *J* = 4.2 Hz, 4H, NCH_2_), 3.72 (t, ^3^ *J* = 4.8 Hz, 4H, NCH_2_), 3.66 (s, 8H, OCH_2_); ^13^C NMR (101 MHz, D_2_O): δ = 169.5 (C=O), 168.5 (C=O), 163.3 (CH_Ar_), 163.0 (C_Ar_), 162.9 (C_Ar_), 161.6 (CH_Ar_), 159.6 (C_Ar_), 158.9 (C_Ar_), 125.7 (CH_Ar_), 123.1 (CH_Ar_), 72.4 (NCH_2_), 66.7 (OCH_2_), 66.6 (OCH_2_), 60.6 (CH_2_Ar), 59.3 (CH_2_Ar), 57.4 (OCH_2_); ^15^N NMR (61 MHz): δ = 285.3 (N′_Ar_), 281.6 (N′_Ar_), 279.3 (N_Ar_), 278.5 (N_Ar_), 46.4 (N_amine_), 45.6 (N_amine_). HRMS (ESI+): *m/z* = 535.2512 [M + H]^+^ (calcd. 535.2511).

### Radiolabeling

*Caution! The selected radionuclides* ^*131*^ *Ba,* ^*133*^ *La,* ^*203*^ *Pb,* ^*212*^ *Pb,* ^*223*^ *Ra,* ^*224*^ *Ra, and* ^*225*^ *Ac represent α, β, and γ emitting radionuclides. Therefore, the following experiments were performed in a controlled area where the handling of radioactive materials is permitted and regulated.*

The radiometals ^131^Ba and ^133^La were in-house produced by cyclotron-assisted proton irradiation of stable CsCl and enriched [^134^Ba]­BaCO_3_, respectively, using a TR-FLEX cyclotron (ACSI). The radiochemical processing of the irradiated targets is described elsewhere.^22,39^ ^131^Ba was formulated as [^131^Ba]­Ba­(NO_3_)_2_ in 0.05 M HNO_3_ while ^133^La as [^133^La]­LaCl_3_ in 0.05 M HCl aqueous solution. Moreover, ^223^Ra was used from a Xofigo solution. ^225^Ac was purchased from ITM Munich (Germany) as a 0.1 M HCl solution and used without further purification. ^203^Pb and ^224^Ra were provided through PRISMAP as NH_4_OAc buffer (pH 5–7) and chloride solution, respectively. Furthermore, ^212^Pb was isolated from its parent nuclide ^224^Ra in an inverse generator based on a crown-ether chromatography resin. Briefly, 180 mg of TK102 (TrisKem, Bruz, France) were packed into an empty column (V = 2 mL) and conditioned with 3 × 0.5 mL of H_2_O and 3 × 0.5 mL of 1 M HCl. The [^224^Ra]­RaCl_2_ was loaded onto this column, washed with 2 × 0.5 mL of 1 M HCl to quantitively recover the ^224^Ra, followed by 6 × 0.5 mL of 1 M HCl to remove the remaining radium traces. The ^212^Pb was then eluted with 4 × 0.5 mL of 1 M NH_4_OAc and directly used for radiolabeling. Radiolabeling was carried out to demonstrate the applicability of the novel chelators for radiopharmaceutical purposes with different radionuclides. Therefore, 100 kBq of ^203^Pb, ^212^Pb, ^133^La, ^225^Ac, and ^131^Ba as well as 10 kBq of ^223^Ra were incubated with different concentrations (10^–3^, 10^–4^, 10^–5^, 10^–6^, 10^–7^, 5·10^–8^ M) of the respective chelator in 0.2 M NH_4_OAc buffer at pH 6 for 1 h at 25 °C. Two TLC systems, normal-phase (NP-TLC) and reversed-phase (RP-TLC), were used to monitor the radiochemical conversion (RCC).^32,39^ The first system (RP-TLC) was designed to quantify the RCC of the radiolabeling reaction and employed an RP-18 TLC plate with an acetonitrile/water (7:3) mobile phase. The second system (NP-TLC) was used to assess complex stability in a competition experiment with EDTA. In this setup, a normal-phase TLC plate and a 50 mM EDTA solution at pH 6 served as the mobile phase.

## Supplementary Material



## Notes

Detailed NMR spectra; HRMS data; ITC data; radiolabeling procedures; computational calculations to determine the structure of the complexes­(DOCX)

## Abbreviations

crown: (2,2′,2″,2‴-(1,10-dioxa-4,7,13,16-tetraazacyclooctadecane-4,7,13,16-tetrayl)­tetraacetic acid
DIPEA: *N,N*-diisopropylethylamine
DOTA: 2,2′,2″,2‴-(1,4,7,10-tetraazacyclododecane-1,4,7,10-tetrayl)­tetraacetic acid
EDTA: ethylenediaminetetraacetic acid
HSAB: hard and soft (Lewis) acids and bases
LET: linear energy transfer
MOPSO: 2-hydroxy-3-(morpholin-4-yl)­propane-1-sulfonic acid
PET: positron emission tomography
PSC: Pb-specific chelator
RCC: radiochemical conversion
SPECT: single photon emission computed tomography
TAT: targeted alpha therapy
TCMC: 1,4,7,10-tetraazacyclododecane-1,4,7,10-tetraacetic amide
TLC: thin layer chromatography
