# Supplementary material for: The Second Nitrogen Fits: Development of Diazine-Based Macrocyclic Chelators to Stably Bind Radionuclides Used in Targeted Alpha Therapy
Source: Inorg Chem. 2026 Sep 16;65(38):22246–60. doi: 10.1021/acs.inorgchem.6c01646 (PMC13625956; doi:10.1021/acs.inorgchem.6c01646)
Supplement: Supplementary file 1 [file ic6c01646_si_001.docx]

**Supporting Information**

The second nitrogen fits: Development of diazine-based macrocyclic chelators to stably bind radionuclides used in targeted alpha therapy

Magdalena K. Blei,^a,b^ Lukas Waurick,^b,c^ Björn Drobot,^c^ Santiago A. Brühlmann,^a^ Charlotte Duchemin,^d^ Edgars Mamis,^d^ Muhammad Inzamam,^d^ Thierry Stora,^d^ Nadia Audouin,^e^ Thomas Sounalet,^f^ Klaus Kopka,^a,b,g,h^ Thorsten Stumpf,^b,c^ Sven Stadlbauer,^a,b^ Jerome Kretzschmar*^c^ and Constantin Mamat*^a,b^

a	Helmholtz-Zentrum Dresden-Rossendorf, Institute of Radiopharmaceutical Cancer Research, Bautzner Landstr. 400, D-01328 Dresden, Germany

b	TU Dresden, School of Science, Faculty of Chemistry and Food Chemistry, D-01062 Dresden, Germany

c	Helmholtz-Zentrum Dresden-Rossendorf, Institute of Resource Ecology, Bautzner Landstr. 400, D-01328 Dresden, Germany

d	CERN, The European Organization for Nuclear Research, CH-1211 Geneva 23, Switzerland.

e	GIP ARRONAX, 1 Rue Aronnax, 44800 Saint-Herblain, France.

f	Subatech, UMR 6457, IMT Atlantique, CNRS/IN2P3, Nantes Université, Nantes, France.

g	National Center for Tumor Diseases (NCT), NCT/UCC Dresden, a partnership between DKFZ, University Hospital Carl Gustav Carus and Faculty of Medicine at TU Dresden, and Helmholtz-Zentrum Dresden-Rossendorf (HZDR), D-01307 Dresden, Germany

h	German Cancer Consortium (DKTK), Partner Site Dresden, Fetscherstr. 74, D-01307 Dresden, Germany

Corresponding authors e-mails: c.mamat@hzdr.de, j.kretzschmar@hzdr.de

Content

[Synthesis Procedures	4](#_Toc239931824)

[Preparation of the complexes for NMR measurements	8](#_Toc239931825)

[NMR spectra of [M(pym-2,4)]^(n−2)+^ complexes	10](#_Toc239931826)

[[Ba(pym-2,4)]	10](#_Toc239931827)

[[La(pym-2,4)]^+^	10](#_Toc239931828)

[[Pb(pym-2,4)]	11](#_Toc239931829)

[NMR spectroscopy of [M(pym-2,6)]^(n−2)+^ complexes	11](#_Toc239931830)

[[Ba(pym-2,6)]	11](#_Toc239931831)

[[La(pym-2,6)]^+^	12](#_Toc239931832)

[[Pb(pym-2,6)]	12](#_Toc239931833)

[NMR spectroscopy of [M(pazi)]^(n−2)+^ complexes	12](#_Toc239931834)

[[Ba(pazi)]	13](#_Toc239931835)

[[La(pazi)]^+^	13](#_Toc239931836)

[[Pb(pazi)]	13](#_Toc239931837)

[NMR spectroscopy of [M(pym-mix)]^(n−2)+^ complexes	14](#_Toc239931838)

[[Ba(pym-mix)]	14](#_Toc239931839)

[[La(pym-mix)]^+^	14](#_Toc239931840)

[[Pb(pym-mix)]	15](#_Toc239931841)

[^1^H NMR spectra for p*K*_a_ determination	16](#_Toc239931842)

[^13^C NMR spectra of the respective complexes	17](#_Toc239931843)

[^15^N NMR spectroscopy	18](#_Toc239931844)

[^207^Pb NMR spectroscopy	19](#_Toc239931845)

[HRMS spectra	20](#_Toc239931846)

[Isothermal Titration Calorimetry (ITC)	26](#_Toc239931847)

[[Ba(pym-2,4)]	28](#_Toc239931848)

[[Pb(pym-2,4)]	29](#_Toc239931849)

[[Ba(pym-2,6)]	30](#_Toc239931850)

[[Pb(pym-2,6)]	31](#_Toc239931851)

[[Ba(pazi)]	32](#_Toc239931852)

[[Pb(pazi)]	33](#_Toc239931853)

[[Ba(pym-mix)]	34](#_Toc239931854)

[[Pb(pym-mix)]	35](#_Toc239931855)

[La-complexes data processing	36](#_Toc239931856)

[[La(pym-2,4)]	37](#_Toc239931857)

[[La(pym-2,6)]	38](#_Toc239931858)

[[La(pazi)]	39](#_Toc239931859)

[[La(pym-mix)]	40](#_Toc239931860)

[Radiolabeling	41](#_Toc239931861)

[Computational Calculations	46](#_Toc239931862)

[Determination of the bond lengths M‒N_arom_	47](#_Toc239931863)

[Determination of the bond lengths M‒O_carbox_	47](#_Toc239931864)

[Determination of the bond lengths M‒N_amine_	48](#_Toc239931865)

[Determination of the bond lengths M‒O_macro_	48](#_Toc239931866)

[Molecular structures of the computed complexes	49](#_Toc239931867)

# Synthesis Procedures

**General Information**

All chemicals used for the syntheses were purchased from commercial suppliers and used without further purification. NMR samples were prepared using deuterated solvents (deutero GmbH) D_2_O (99.95% D) and D_2_O solutions of both DCl (38% in D_2_O with 99% D) and NaOD (40% in D_2_O with 99% D). pD values were measured using a pH meter (VWR pHenomenal MU 6100 L) with a pH electrode (WTW SenTix Mic) and corrected using the formula: *pD = pH + 0.44*.^63^ A 1 mM stock solution of each chelator with 0.1 M NaCl was prepared in D_2_O, with pD adjusted between 0.7 and 11.3 in 1 mL volumes. High-purity metals (99.99%) were weighed with plastic spatulas. For NMR experiments, 0.1 M metal salt stock solutions (La^3+^, Pb^2+^, Ba^2+^) were prepared in D_2_O. Samples used for ligand characterization were prepared with 10 mM of ligand at pD 4. Samples aiming at structure elucidation of metal chelates were prepared with a ligand concentration of 12.5 mM and a metal ion concentration of 15 mM at pD 6. For ITC experiments, metal salts were dissolved in Milli-Q water (Merck Millipore). ITC measurements were performed at 25 °C with 0.1 M NaCl ionic strength.

The synthesis of macropa^32,39^ as well as the synthesis of the aromatic side-arms **2a** and **2b**^52^ is reported elsewhere. The general synthesis steps are shown in Figure 2, and the corresponding analytical data are given in the SI.

**Methyl 6-(bromomethyl)pyrazine-2-carboxylate (2c).** Methyl 6-methylpyrazine-2-carboxylate (1.23 g, 8.08 mmol) and *N*-bromosuccinimide (1.29 g, 7.28 mmol) were dissolved in CCl_4_ (10 mL) and the solution was then heated to 80 °C. Afterwards, azobis(isobutyronitrile) (398 mg, 2.43 mmol) was added and the reaction was stirred at 80 °C for 16 h. After cooling to rt, the solvent was removed and the crude product was purified by automated column chromatography (silica gel, 0/100 🡪 40/100 petroleum ether/ethyl acetate). Compound **2c** was obtained in 19% (351 mg) yield as a yellow oil. Additionally, 529 mg of the starting material (conversion: 57 %) were recovered and the double-brominated by-product (320 mg, 13%) was obtained as well. ^1^H NMR (400 MHz, CDCl_3_): *δ* = 9.17 (d, ^4^*J* = 0,6 Hz, 1H, H_Ar_), 8.89 (d, ^4^*J* = 0,6 Hz, 1H, H_Ar_), 4.60 (s, 2H, CH_2_Ar), 4.00 (s, 3H, CH_3_) ppm. ^13^C NMR (101 MHz, CDCl_3_): *δ* = 164.02 (C=O), 152.53 (C_Ar_), 148.01 (CH_Ar_), 144.93 (CH_Ar_), 142.15 (C_Ar_), 53.26 (CH_3_) 29.47 (CH_2_Ar) ppm. MS (ESI+): *m/z* = 231 [M + H]^+^ (^79^Br), 233 [M + H]^+^ (^81^Br).

**General synthesis of the macrocycle with the side arms.** Kryptofix 2.2 (**1**) (1 eq.), *N*,*N*-diisopropylethylamine (3 eq.) and the respective methyl 6-(bromomethyl)(diazine)-carboxcylates **2a-c** (2.1 eq.) were dissolved in anhydrous acetonitrile and the mixture was stirred at 75 °C for 16 h. After cooling to rt, the solvent was removed and the crude product was purified by automated column chromatography (silica gel, 0/100 🡪 20/100 ethyl acetate + 1 % NEt_3_ / ethanol + 1 % NEt_3_). Compounds **3a-c** were obtained as yellow oils.

**Dimethyl 2,2'-((1,4,10,13-tetraoxa-7,16-diazacyclooctadecane-7,16-diyl)bis-(methylene))-bis(pyrimidine-4-carboxylate) (3a).** Methyl 2-(bromomethyl) pyrimidine-4-carboxylate (**2a**, 380 mg, 1.65 mmol), Kryptofix 2.2 (**1**, 173 mg, 0.66 mmol) and DIPEA (345 µL, 1.98 mmol) were reacted in 15 mL of acetonitrile to obtain **3a** in a yield of 250 mg (67%). ^1^H NMR (400 MHz, CDCl_3_): *δ* = 8.94 (dd, ^3^*J* = 5.0 Hz, ^4^*J* = 1.1 Hz, 2H, H_Ar_), 7.81 (d, ^3^*J* = 4.9 Hz, 2H, H_Ar_), 4.18 (s, 4H, CH_2_Ar), 3.99 (s, 6H, CH_3_), 3.67 (t, ^3^*J* = 5.7, 8H, OCH_2_), 3.57 (s, 8H, OCH_2_), 3.02 (t, ^3^*J* = 5.7 Hz, 8H, NCH_2_) ppm; ^13^C NMR (101 MHz, CDCl_3_): *δ* = 169.5 (C_Ar_), 164.8 (C=O), 159.2 (CH_Ar_), 154.8 (C_Ar_), 118.6 (CH_Ar_), 70.6 (OCH_2_), 69.7 (OCH_2_), 61.3 (CH_2_Ar), 53.8 (NCH_2_), 53.3 (CH_3_) ppm. MS (ESI+): *m/z* = 562 [M]^+^.

**Dimethyl 4,4'-((1,4,10,13-tetraoxa-7,16-diazacyclooctadecane-7,16-diyl)bis(methylene))-bis(pyrimidine-2-carboxylate) (3b).** Methyl 6-(bromomethyl)pyrazine-2-carboxylate (**2b**, 380 mg, 1.64 mmol), Kryptofix 2.2 (**1**, 205 mg, 0.78 mmol) and DIPEA (409 µL, 2.35 mmol) were reacted in 15 mL of acetonitrile to obtain **3b** in a yield of 351 mg (80%). ^1^H NMR (400 MHz, CDCl_3_): *δ* = 8.83 (d, ^3^*J* = 5.1 Hz, 2H, CH_Ar_), 8.04 (d, ^3^*J* = 5.1 Hz, 2H, CH_Ar_), 4.04 (s, 6H, CH_3_), 3.96 (s, 4H, CH_2_Ar), 3.56 (t, ^3^*J* = 5.4 Hz, 8H, OCH_2_), 3.49 (s, 8H, OCH_2_), 2.88 (t, ^3^*J* = 5.4 Hz, 8H, NCH_2_) ppm; ^13^C NMR (151 MHz, CDCl_3_): *δ* = 172.0 (C_Ar_), 164.2 (C=O), 158.1 (CH_Ar_), 155.8 (C_Ar_), 122.2 (CH_Ar_), 70.8 (OCH_2_), 69.7 (OCH_2_), 60.7 (CH_2_Ar), 55.4 (NCH_2_), 53.7 (CH_3_) ppm. MS (ESI+): *m/z* = 562 [M]^+^.

**Dimethyl 6,6'-((1,4,10,13-tetraoxa-7,16-diazacyclooctadecane-7,16-diyl)bis(methylene))-bis(pyrazine-2-carboxylate) (3c).** Methyl 4-(bromomethyl)pyrimidine-2-carboxylate (**2c**, 205 mg, 0.89 mmol), Kryptofix 2.2 (**1**, 93 mg, 0.35 mmol) and DIPEA (186 µL, 1.06 mmol) were reacted in 10 mL of acetonitrile to obtain **3c** in a yield of 185 mg (79%). ^1^H NMR (400 MHz, CDCl_3_): *δ* = 9.14 (s, 2H, CH_Ar_), 9.12 (s, 2H, CH_Ar_), 4.02 (s, 4H, CH_2_Ar), 4.00 (s, 6H, CH_3_), 3.62 (t, ^3^*J* = 5.6 Hz, 8H, OCH_2_), 3.56 (s, 8H, OCH_2_), 2.88 (t, ^3^*J* = 5.6 Hz, 8H, NCH_2_) ppm; ^13^C NMR (101 MHz, CDCl_3_): *δ* = 164.7 (C=O), 156.3 (C_Ar_), 148.7 (CH_Ar_), 144.1 (CH_Ar_), 141.5 (C_Ar_), 70.7 (OCH_2_), 69.8 (OCH_2_), 59.6 (CH_2_Ar), 54.69 (NCH_2_), 53.02 (OCH_3_) ppm. MS (ESI+): *m/z* = 562 [M]^+^.

**Methyl 4-((16-((4-(methoxycarbonyl)pyrimidin-2‑yl)methyl)-1,4,10,13-tetraoxa-7,16-diaza-cyclooctadecan-7-yl)methyl) pyrimidine-2‑carboxylate (3d).** Kryptofix 2.2 (1, 2 eq., 175 mg, 0.67 mmol) and DIPEA (3 eq., 174 µL, 1.00 mmol) were dissolved in 50 mL of anhydrous acetonitrile and the mixture was heated to 75°C. Methyl 4-(bromomethyl)pyrimidine-2-carboxylate **2b** (1 eq., 77 mg, 0.33 mmol) dissolved in 20 mL of anhydrous acetonitrile was added over 5 h using a syringe pump and the mixture was finally stirred for 16 h at 75°C. After cooling to rt, the solvent was removed and the crude product was purified by automated column chromatography (silica gel, 0/100 🡪 20/100 ethyl acetate + 1 % NEt_3_ / ethanol + 1 % NEt_3_). The resulting methyl 4-((1,4,10,13-tetraoxa-7,16-diazacyclooctadecan-7-yl) methyl)pyrimidine-2-carboxylate was obtained (89 mg, 65%) as yellow oil. ^1^H NMR (600 MHz, CDCl_3_): *δ* = 8.90 (d, ^3^*J* = 5.1 Hz, 1H, CH_Ar_), 7.86 (d, ^3^*J* = 5.1 Hz, 1H, CH_Ar_), 3.94 (s, 3H, CH_3_), 3.86 (s, 2H, CH_2_Ar), 3.82 (t, ^3^*J* = 4.6 Hz, 4H, OCH_2_), 3.53–3.49 (m, 4H, OCH_2_), 3.46 (t, ^3^*J* = 4.8 Hz, 4H, OCH_2_), 3.43–3.38 (m, 4H, OCH_2_), 3.14 (t, ^3^*J* = 4.8 Hz, 4H, NCH_2_), 2.76 (t, ^3^*J* = 4.8 Hz, 4H, NCH_2_) ppm; ^13^C NMR (151 MHz, CDCl_3_): *δ* = 171.0 (C_Ar_), 164.0 (C=O), 158.1 (CH_Ar_), 155.4 (C_Ar_), 122.3 (CH_Ar_), 69.8 (OCH_2_), 68.4 (OCH_2_), 65.9 (OCH_2_), 58.8 (CH_2_Ar), 56.1 (NCH_2_), 53.4 (CH_3_), 48.3 (NCH_2_) ppm. MS (ESI+): *m/z* = 413 [M+H]^+^ (calculated: 414). Secondly, this intermediate compound (1 eq., 89 mg, 0.22 mmol), DIPEA (3 eq., 113 µL, 0.65 mmol) and methyl 2‑(bromomethyl) pyrimidine-4-carboxylate **2a** (63 mg, 0.27 mmol) were dissolved in 10 mL of anhydrous acetonitrile and stirred at 75°C for 16 h. After cooling to rt, the solvent was removed and the crude product was purified by automated column chromatography (silica gel, 0/100 🡪 20/100 ethyl acetate + 1 % NEt_3_ / ethanol + 1 % NEt_3_) to obtain **3d** (64 mg, 53 %) as yellow oil. ^1^H NMR (400 MHz, CDCl_3_): *δ* = 8.95 (d, ^3^*J* = 5.0 Hz, 1H, H5), 8.84 (d, ^3^*J* = 5.1 Hz, 1H, H6’), 8.02 (d, ^3^*J* = 5.1 Hz, 1H, H4), 7.83 (d, ^3^*J* = 5.0 Hz, 1H, H4), 4.20 (s, 2H, H10), 4.03 (s, 3H, H20), 4.00 (s, 3H, H20’), 3.96 (s, 2H, H10’), 3.68 (t, ^3^*J* = 5.8 Hz, 4H, H18), 3.57 (p, ^3^*J* = 5.5 Hz, 12H, H13+15+16), 3.05 (s, 4H, H19), 2.85 (t, ^3^*J* = 5.4 Hz, 4H, H12) ppm. ^13^C NMR (101 MHz, CDCl_3_): *δ* = 172.05 (C7’), 169.36 (C7), 164.85 (C3), 164.26 (C3’), 159.40 (C5’), 158.05 (C6), 155.80 (C1), 154.98 (C1’), 122.16 (C5’), 118.82 (C4), 70.88 (C15), 70.68 (C16), 69.85 (C18), 69.73 (C13), 61.19 (C10), 60.92 (C10’), 55.16 (C12), 54.09 (C19), 53.61 (C20), 53.48 (C20’) ppm. MS (ESI+): *m/z* = 563 [M+H]^+^.

**General saponification procedure.** The bis(methylesters) **3a-d** (1 eq.) and lithium hydroxide (6 eq.) were dissolved in a 1:1 methanol/water solution and stirred at 50 °C overnight. After cooling to rt, the solvent was removed and the crude product was purified by semi-preparative HPLC (Agilent Zorbax SB C-18 5 µm 80 Å, 9.4 × 250 mm, flow rate: 6 mL/min, gradient: 5/95 🡪 40/60 acetonitrile/water + 0.1 % TFA in 40 min). All chelators were obtained as colorless solids.

***N,N′*-Bis[(4-carboxy-2-pyrimidinyl)methyl]-1,10-diaza-18-crown-6 (pym-2,4).** LiOH (77 mg, 3.2 mmol) and **3a** (300 mg, 0.53 mmol) were reacted in 10 mL of a 1:1 methanol/water solution to yield 80 mg (28%) of **pym‑2,4** after HPLC-purification. ^1^H NMR (400 MHz, D_2_O): *δ* = 8.80 (d, ^3^*J* = 5.2 Hz, 2H, CH_Ar_), 7.68 (d, ^3^*J* = 5.2 Hz, 2H, CH_Ar_), 4.68 (s, 4H, CH_2_Ar), 3.79–3.70 (m, 8H, O-CH_2_), 3.54 (t, ^3^*J* = 4.9 Hz, 8H, NCH_2_), 3.36 (s, 8H, OCH_2_) ppm; ^13^C NMR (101 MHz, D_2_O): *δ* = 170.1 (C_Ar_), 161.3 (C=O), 160.0 (C_Ar_), 159.7 (CH_Ar_), 119.3 (CH_Ar_), 69.5 (OCH_2_), 64.0 (OCH_2_), 58.5 (CH_2_Ar), 54.8 (NCH_2_) ppm; ^15^N NMR (61 MHz): *δ* = 285.2 (N’_arom_), 279.0 (N_arom_), 46.5 (N_amine_) ppm. HRMS (ESI+): *m/z* = 535.2509 [M+H]^+^ (calcd. 535.2511).

***N,N′*-Bis[(2-carboxy-4-pyrimidinyl)methyl]-1,10-diaza-18-crown-6  (pym-2,6).** Compound **3c** (190 mg, 0.34 mmol) and LiOH (49 mg, 2.05 mmol) were reacted in a 1:1 methanol/water solution to yield 104 mg (57%) of **pym-2,6** after purification. ^1^H NMR (400 MHz, D_2_O): *δ* = 8.77 (d, ^3^*J* = 5.2 Hz, 2H, CH_Ar_), 7.53 (d, ^3^*J* = 5.2 Hz, 2H, CH_Ar_), 4.67 (s, 4H, CH_2_Ar), 3.80 (t, ^3^*J* = 4.5 Hz, 8H, OCH_2_), 3.55 (t, ^3^*J* = 4.9 Hz, 8H, NCH_2_), 3.49 (s, 8H, OCH_2_) ppm; ^13^C NMR (101 MHz, D_2_O): *δ* = 169.8 (C=O), 161.0 (C_Ar_), 159.6 (C_Ar_), 158.6 (CH_Ar_), 121.2 (CH_Ar_), 69.7 (OCH_2_), 64.0 (OCH_2_), 57.1 (CH_2_Ar), 54.6 (NCH_2_) ppm; ^15^N NMR (61 MHz): *δ* = 281.6 (N’_arom_), 278.4 (N_arom_), 45.6 (N_amine_) ppm. HRMS (ESI+): *m/z* = 535.2510 [M+H]^+^ (calcd. 535.2511).

***N,N′*-Bis[(2-carboxy-6-pyrazinyl)methyl]-1,10-diaza-18-crown-6 (pazi).** LiOH (12 mg, 0.5 mmol) and **3b** (35 mg, 0.062 mmol) were reacted in 5 mL of a 1:1 methanol/water solution to yield 31 mg (92%) of **pazi** after HPLC-purification. ^1^H NMR (400 MHz, D_2_O): *δ* = 8.99 (s, 2H, CH_Ar_), 8.69 (s, 2H, CH_Ar_), 4.69 (s, 4H, CH_2_Ar), 3.82–3.77 (m, 8H, OCH_2_), 3.55 (t, ^3^*J* = 4.9 Hz, 8H, NCH_2_), 3.49 (s, 8H, OCH_2_) ppm; ^13^C NMR (101 MHz, D_2_O): *δ* = 169.8 (C=O), 149.5 (CH_Ar_), 148.2 (C_Ar_), 147.8 (CH_Ar_), 147.3 (C_Ar_), 72.3 (OCH_2_), 66.4 (OCH_2_), 58.0 (CH_2_Ar), 56.9 (NCH_2_) ppm; ^15^N NMR (61 MHz): *δ* = 325.1 (N’_arom_), 323.8 (N_arom_), 47.1 (N_amine_) ppm. HRMS (ESI+): *m/z* = 535.2511 [M+H]^+^ (calcd. 535.2511).

***N*-[(2-carboxy-4-pyrimidinyl)methyl]-*N′*-[(4-carboxy-2-pyrimidinyl)methyl]-1,10-diaza-18-crown-6 (pym-mix).** Compound **3d** (52 mg, 0.09 mmol) and LiOH (14 mg, 0.55 mmol) were reacted in 4 mL of a 1:1 methanol/water solution to yield 32 mg (65%) of **pym-mix** after HPLC-purification. ^1^H NMR (400 MHz, D_2_O): *δ* = 9.11 (d, ^3^*J* = 5.1 Hz, 1H, CH_Ar_), 9.02 (d, ^3^*J* = 5.2 Hz, 1H, CH_Ar_), 8.07 (d, ^3^*J* = 5.1 Hz, 1H, CH_Ar_), 7.83 (d, ^3^*J* = 5.2 Hz, 1H, CH_Ar_), 4.92 (s, 2H, CH_2_Ar), 4.89 (s, 2H, CH_2_Ar), 4.00–3.93 (m, 8H, OCH_2_), 3.76 (t, ^3^*J* = 4.2 Hz, 4H, NCH_2_), 3.72 (t, ^3^*J* = 4.8 Hz, 4H, NCH_2_), 3.66 (s, 8H, OCH_2_); ^13^C NMR (101 MHz, D_2_O): *δ* = 169.5 (C=O), 168.5 (C=O), 163.3 (CH_Ar_), 163.0 (C_Ar_), 162.9 (C_Ar_), 161.6 (CH_Ar_), 159.6 (C_Ar_), 158.9 (C_Ar_), 125.7 (CH_Ar_), 123.1 (CH_Ar_), 72.4 (NCH_2_), 66.7 (OCH_2_), 66.6 (OCH_2_), 60.6 (CH_2_Ar), 59.3 (CH_2_Ar), 57.4 (OCH_2_); ^15^N NMR (61 MHz): *δ* = 285.3 (N’_arom_), 281.6 (N’_arom_), 279.3 (N_arom_), 278.5 (N_arom_), 46.4 (N_amine_), 45.6 (N_amine_). HRMS (ESI+): *m/z* = 535.2512 [M+H]^+^ (calcd. 535.2511).

# Preparation of the complexes for NMR measurements

In general, NMR data acquisition was according to the procedures and setups described in detail elsewhere.^[1]^ Briefly, spectra were recorded at (25 ± 0.2) °C on Agilent DD2-600 and 400 MR systems operating at 14.1 and 9.4 T, with resonance frequencies of 599.8 and 399.8 MHz for ^1^H and 150.9 and 100.6 MHz for ^13^C, respectively, using 5 mm NMR probes. Additionally, ^15^N and ^207^Pb NMR spectra were recorded at 60.8 and 125.5 MHz, respectively (at 14.1 T). Chemical shifts are reported in parts per million (ppm) relative to external TMS (0 ppm for both ^1^H and ^13^C), liquid ammonia (0 ppm for ^15^N) and PbMe_4_ (0 ppm for ^207^Pb, using Pb(NO_3_)_2_ in D_2_O as secondary reference), respectively. ^15^N NMR chemical shifts were obtained from the 2D ^1^H^15^N-HMBC experiment. NMR spectra were processed with *MestReNova*, version 14.2.3 (Mestrelab Research SL). The generation of graphs for the purpose of numerical data visualization and data fitting by means of a nonlinear sigmoidal dose–response fit algorithm was performed utilizing *Origin 2020*, version 9.7.0.185 (OriginLab Corp.).

A stock solution of the respective chelator (1 equiv.) was prepared in D_2_O and mixed with a stock solution of the desired metal salt (1.2 equiv.) in D_2_O. The pH is then adjusted to 6 using DCl and NaOD and the sample was centrifuged, and the supernatant solution was analyzed by NMR spectroscopy.

Table S1. Composition of the stock solutions of the metal salts used for NMR spectroscopy.

| Metal salt | M (g/mol) | m (mg) | D_2_O in mL | c (mol/L) |
| --- | --- | --- | --- | --- |
| La(NO_3_)_3_ ‧ 6 H_2_O | 433.02 | 77.9 | 1.798 | 0.1 M |
| BaCl_2_ ‧ 2 H_2_O | 244.26 | 27.3 | 1.119 | 0.1 M |
| Pb(NO_3_)_2_ ‧ 6 H_2_O | 331.05 | 40.3 | 1.216 | 0.1 M |

Table S2. Composition of the stock solutions of the chelators used for NMR spectroscopy.

| Chelator | M (g/mol) | m (mg) | D_2_O in mL | c (mmol/L) |
| --- | --- | --- | --- | --- |
| pym-2.4 ‧ 2 TFA | 762.6 | 28.9 | 1.514 | 25 mM |
| pym-2.6 ‧ 2 TFA | 762.6 | 30.07 | 1.577 | 25 mM |
| pazi ‧ 2 TFA | 762.6 | 29.2 | 1.532 | 25 mM |
| pym-mix ‧ 2 TFA | 762.6 | 23.25 | 1.525 | 20 mM |

Table S3. General contribution of the complexes of the different stock solutions.

| Chelator | Stock solution chelator | Stock solution metal | D_2_O + pH/pD |
| --- | --- | --- | --- |
| pym-2,4  pym-2,6  pazi | 300 µL | 90 µL | 210 µL |
| pym-mix | 240 µL | 72 µL | 288 µL |

# NMR spectra of [M(pym-2,4)]^(n−2)+^ complexes

## [Ba(pym-2,4)]

^1^H NMR (600 MHz, D_2_O): *δ* = 8.68 (dd, ^3^*J* = 5.1, ^4^*J* = 1,2 Hz, 2H, H5), 7.35 (d, ^3^*J* = 5.1 Hz, 2H, H6), 4.79 (d, ^2^*J* = 16.5 Hz, 2H, H10a), 4.19 (t, ^3^*J* = 11.6 Hz, 2H, H13a), 3.91 (t, ^3^*J* = 11.4 Hz, 2H, H18a), 3.87 (t, ^3^*J* = 10.8 Hz, 2H, H16a), 3.81 (t, ^3^*J* = 11.2 Hz, 2H, H15a), 3.50 (d, ^3^*J* = 9,9 Hz, 2H, H13a), 3.47 (d, ^2^*J* = 16.2 Hz, 2H, H10b), 3.44 (s, 2H, H15b), 3.41 (d, ^3^*J* = 10.2 Hz, 2H, H16b), 3.27 (t, ^2^*J* = 13.0 Hz, 2H, H12a), 3.23 (d, ^3^*J* = 10.9 Hz, 2H, H18b), 2.59 (t, ^3^*J* = 13.2 Hz, 2H, H19a), 2.35 (d, ^3^*J* = 14.0 Hz, 2H, H12b), 2.28 (d, ^3^*J* = 14.3 Hz, 2H, H19b) ppm; ^13^C NMR (151 MHz, D_2_O): *δ* = 168.95 (C1), 167.60 (C7), 159.68 (C5), 159.50 (C3), 118.07 (C6), 70.31 (C16), 69.70 (C15), 67.43 (C18), 67.33 (C13), 59.48 (C10), 55.70 (C19), 53.15 (C12) ppm; ^15^N NMR (^1^H^15^N HMBC, 61 MHz, D_2_O): *δ* = 284.0 (N4), 278.4 (N2), 40.2 (N11) ppm.

## [La(pym-2,4)]^+^

^1^H NMR (600 MHz, D_2_O): *δ* = 8.80 (d, ^3^*J* = 5.1 Hz, 2H, H5), 7.36 (d, ^3^*J* = 5.1 Hz, 2H, H6), 5.12 (d, ^2^*J* = 16.5 Hz, 2H, H10a), 4,33 (ddd, ^3^*J* = 12.7, ^3^*J* = 10.4, ^4^*J* = 1.9 Hz, 2H, H13a), 4.01 (t, ^3^*J* = 9.8 Hz, 2H, H18a), 3.98 (dd, ^3^*J* = 10.0, ^4^*J* = 2.7 Hz, 2H, H16a), 3.89 (ddd, ^2^*J* = 12.3, ^3^*J =* 10.5, ^4^*J =* 2.7 Hz, 2H, H15a), 3.66 (dd, ^3^*J* = 10.4, ^4^*J* = 3,3 Hz, 2H, H13b), 3.57 (s, 2H, H10b), 3.57 – 5.54 (m, 2H, H15b), 3.54 – 3.51 (m, 2H, H16b) 3.40 (td, ^3^*J* = 13.4, ^4^*J* = 3.7 Hz, 2H, H12a), 3.38 – 3.33 (m, 2H, H18b), 2.64 (ddd, ^3^*J* = 15.2, ^3^*J* = 12.3, ^4^*J* = 3.4 Hz, 2H, H19a), 2.53 (dt, ^2^*J* = 14.0, ^4^*J =* 1.7 Hz, 2H, H12b), 2.39 (d, ^2^*J* = 14.1 Hz, 2H, H19b) ppm; ^13^C NMR (151 MHz, D_2_O): *δ* in ppm = 169.67 (C7), 167.91 (C3), 161.45 (C5), 156.99 (C1), 117.55(C6), 71.39 (C16), 69.68 (C15), 68.63 (C18), 67.70 (C13), 60.52 (C10), 55.84 (C19), 52.93 (C12) ppm; ^15^N NMR (^1^H^15^N HMBC, 61 MHz, D_2_O): *δ* in ppm = 288.9 (N4), 269.6 (N2), 46.5 (N11) ppm.

## [Pb(pym-2,4)]

^1^H NMR (600 MHz, D_2_O): *δ* = 8.86 (d, ^3^*J* = 5.0 Hz, 2H, H5), 7.39 (d, ^3^*J* = 5,0 Hz, 2H, H6), 5.03 (d, ^2^*J* = 16.5 Hz, 2H, H10a), 4.30 (t, ^2^*J* = 11.1 Hz, 2H, H13a), 3.87 (d, ^3^*J* = 6.0 Hz, 2H, H18a), 3.85 (d, ^2^*J* = 12.0 Hz, 2H, H10b), 3.74 (td, ^2^*J* = 10.6, ^4^*J* = 1.6 Hz, 2H, H16a), 3.67 (td, ^2^*J* = 10.6, ^4^*J* = 1.6 Hz, 2H, H15a), 3.60 (dt, ^3^*J* = 4.7, ^4^*J* = 2.2 Hz, 2H, H15b), 3.58 (d, ^4^*J* = 3.4 Hz, 2H, H13b), 3.47 (dd, ^2^*J* = 10,9, ^4^*J* = 2.2 Hz, 2H, H16b), 3.37 (dt, ^2^*J* = 10.9, ^4^*J* = 2.6 Hz, 2H, H18b), 3.18 (ddd, ^2^*J* = 14.7, ^2^*J* = 11.8, ^4^*J* = 3.0 Hz, 2H, H19a), 2.54 (d, ^2^*J* = 14.4 Hz, 2H, H19b), 2.49 – 2.44 (m, 2H, H12a), 2,44 (d, ^4^*J* = 2.7 Hz, 2H, H12b) ppm; ^13^C NMR (151 MHz, D_2_O): *δ* = 169.77 (C7), 168.82 (C3), 158.44 (C5), 157.65 (C1), 123.20 (C6), 70.11 (C16), 69.45 (C15), 68.40 (C18), 67.22 (C13), 58.95 (C10), 55.01 (C19), 53.31 (C12) ppm; ^15^N NMR (^1^H^15^N HMBC, 61 MHz, D_2_O): *δ* = 288.1 (N4), 279.6 (N2), 46.6 (N11) ppm; ^207^Pb (126 MHz, D_2_O): *δ* = -2258 ppm.

# NMR spectroscopy of [M(pym-2,6)]^(n−2)+^ complexes

## [Ba(pym-2,6)]

^1^H NMR (600 MHz, D_2_O): *δ* = 8.54 (d, ^3^*J* = 5.2 Hz, 2H, H5), 7.38 (d, ^3^*J* = 5.2 Hz, 2H, H4), 4.76 (d, ^2^*J* = 16.6 Hz, 2H, H10a), 4.18 (s, 2H, H13a), 3.86 (d, ^3^*J* = 13.4 Hz, 6H, H18a, H16a, H15a), 3.48 (d, ^2^*J* = 9.0 Hz, 2H, H13b), 3.44 (d, ^3^*J* = 9.8 Hz, 2H, H15b), 3.40 (d, ^2^*J* = 12.7 Hz, 4H, H10b, H16b), 3.26 (s, 2H, H12a), 3.21 (d, ^3^*J* = 10.6 Hz, 2H, H18b), 2.56 (s, 2H, H19a), 2.33 (d, ^2^*J* = 14.1 Hz, 2H, H12b), 2.22 (d, ^3^*J* = 14.4 Hz, 2H, H19b) ppm; ^13^C NMR (151 MHz, D_2_O): *δ* = 168.63 (C1), 168.39 (C7), 159.09 (C5), 157.60 (C3), 121.50 (C4), 70.30 (C16), 69.71 (C15), 67.47 (C18), 67.24 (C13), 57.98 C10), 55.37 (C19), 53.35 (C12) ppm; ^15^N NMR (^1^H^15^N HMBC, 61 MHz, D_2_O): *δ* = 279.8 (N2), 279.3 (N6) ppm.

## [La(pym-2,6)]^+^

^1^H NMR (600 MHz, D_2_O): *δ* = 8.65 (d, ^3^*J* = 5.2 Hz, 2H, H5), 7.56 (d, ^3^*J* = 5.2 Hz, 2H, H4), 5.08 (d, ^2^*J* = 16.6 Hz, 2H, H10a), 4.31 (ddd, ^3^*J* = 12.6, ^3^*J* = 10.2, ^4^*J* = 1.8 Hz, 2H, H13a), 4.00 (d, ^3^*J* = 11.2 Hz, 2H, H18a), 3.98 – 3.95 (m, 2H, H16a), 3.89 (ddd, ^3^*J* = 12.4, ^3^*J* = 10.5, ^4^*J* = 2.6 Hz, 2H, H15a), 3.65 (tt, ^3^*J* = 10.3, ^4^*J* = 2.6 Hz, 2H, H13b), 3.54 (d, ^2^*J* = 16.8 Hz, 2H, H10b), 3.54-3, (m, 2H, H15b), 3.53 – 3.49 (m, 2H, H16b), 3.40 (td, ^3^*J* = 13.4, ^4^*J* = 3.7 Hz, 2H, H12a), 3.34 (tt, ^3^*J* = 10.3, ^4^*J* = 2.6 Hz, 2H, H18b), 2.62 (ddd, ^2^*J* = 15.3, ^3^*J* = 12.5, ^4^*J* = 3.4 Hz, 2H, H19a), 2.50 (d, ^3^*J* = 14.2 Hz, 2H, H12b), 2.34 (d, ^3^*J* = 14.1 Hz, 2H, H19b) ppm; ^13^C NMR (151 MHz, D_2_O): *δ* = 168.86 (C1), 168.27 (C7), 159.44 (C5), 157.04 (C3), 122.43 (C4), 71.37 (C16), 69.63 (C15), 68.60 (C18), 67.57 (C13), 59.01 (C10), 55.61 (C19), 53.09 (C12) ppm; ^15^N NMR (^1^H^15^N HMBC, 61 MHz, D_2_O): *δ* = 280.8 (N6), 273.1 (N2), 47.4 (N11) ppm.

## [Pb(pym-2,6)]

^1^H NMR (600 MHz, D_2_O): *δ* = 8.64 (d, ^3^*J* = 5.1 Hz, 2H, H5), 7.68 (d, ^3^*J* = 5.2 Hz, 2H, H4), 5.03 – 4.96 (m, 2H, H10a), 4.27 (t, ^3^*J* = 11.2 Hz, 2H, H13a), 3.85 (dt, ^3^*J* = 13.3, ^3^*J* = 7.1 Hz, 2H, H18a), 3.81 – 3.75 (m, 2H, H10b), 3.72 (t, ^3^*J* = 10.5 Hz, 2H, H16a), 3.66 (t, ^3^*J* = 10.6 Hz, 2H, H15a), 3.59 (d, ^4^*J* = 3.8 Hz, 2H, H15b), 3.57 (d, ^4^*J* = 3.4 Hz, 2H, H13b), 3.46 (d, ^3^*J* = 10.8 Hz, 2H, H16b), 3.35 (dt, ^3^*J* = 10.8, ^4^*J* = 2.6 Hz, 2H, H18b), 3.18 (ddd, ^3^*J* = 14.8, ^3^*J* = 11.8, ^4^*J* = 3.0 Hz, 2H, H12a), 2.53 (d, ^3^*J* = 14.4 Hz, 2H, H12b), 2.42 – 2.37 (m, 4H, H19) ppm; ^13^C NMR (151 MHz, D_2_O): *δ* = 169.77 (C1), 168.82 (C7), 158.44 (C5), 157.65 (C3), 123.20 (C4), 70.11 (C316), 69.45 (C15), 68.40 (C18), 67.22 (C13), 58.95 (C10), 55.01 (C19), 53.31 (C12) ppm; ^15^N NMR (^1^H^15^N HMBC, 61 MHz, D_2_O): *δ* = 283.1 (N6), 283.0 (N2), 47.1 (N11) ppm; ^207^Pb (126 MHz, D_2_O): *δ* = -2305 ppm.

# NMR spectroscopy of [M(pazi)]^(n−2)+^ complexes

## [Ba(pazi)]

^1^H NMR (600 MHz, D_2_O): *δ* = 8.53 (s, 2H, H4), 8.51 (s, 2H, H6), 4.76 (d, ^2^*J* = 16.1 Hz, 2H, H10a), 4.19 (s, 2H, H13a), 3.89 (s, 2H, H18a), 3.85 (d, ^3^*J* = 10.6 Hz, 2H, H16a), 3.81 (d, ^3^*J* =11.9 Hz, 2H, H15a), 3.50 (s, 2H, H13b), 3,48 (s, 2H, H10b) 3.44 (d, ^3^*J* = 10.3 Hz, 2H, H15b), 3.41 – 3.37 (m, 2H, H16b), 3.28 (s, 2H, H19a), 3.22 – 3.18 (m, 2H, H18b), 2.54 (d, ^2^*J* = 13.4 Hz, 2H, H12a), 2.37 (d, ^2^*J* = 14.0 Hz, 2H, H19b), 2.24 (d, ^2^*J* = 14.0 Hz, 2H, H12b) ppm; ^13^C NMR (151 MHz, D_2_O): *δ* = 169.27 (C3), 153.86 (C1), 146.23 (C7), 145.41 (C4), 142.04 (C6), 70.32 (C16), 69.70 (C15), 67.46 (C18), 67.34 (C13), 56.06 (C10), 55.24 (C19), 53.50 (C12) ppm; ^15^N NMR (^1^H^15^N HMBC, 61 MHz, D_2_O): *δ* = 323.7 (N5), 317.5 (N2) ppm.

## [La(pazi)]^+^

^1^H NMR (600 MHz, D_2_O): *δ* = 8.74 (s, 2H, H4 ), 8.54 (s, 2H, H6), 5.07 (d, ^2^*J* = 16.0 Hz, 2H, H10a), 4.32 (ddd, ^3^*J* = 12.5, ^3^*J* = 10.3, ^4^*J* = 1.9 Hz, 2H, H13a), 4.02 – 3.97 (m, 2H, H18a), 3.97 – 3.93 (m, 2H, H16a), 3.86 (ddd,^3^*J* = 12.4, ^3^*J* = 10.6, ^4^*J* = 2.6 Hz, 2H, H15a), 3.68 – 3.62 (m, 2H, H13b), 3.61 (d, ^2^*J* = 16.0 Hz, 2H, H10b), 3.55 – 3.52 (m, 2H, H15b), 3.52 – 3.48 (m, 2H, H16b), 3.45 – 3.37 (m, 2H, H19a), 3.32 (ddd, ^3^*J* = 10.8, ^4^*J* = 3.5, ^4^*J* =1.5 Hz, 2H, H18b), 2.60 (ddd, ^2^*J* = 15.5, ^3^*J* = 12.6, ^4^*J* = 3.5 Hz, 2H, H12a), 2.56 – 2.51 (m, 2H, H19b), 2.37 – 2.32 (m, 2H, H12b) ppm; ^13^C NMR (151 MHz, D_2_O): *δ* = 170.06 (C7), 153.45 (C3), 147.37 (C4), 143.47 (C1), 142.48 (C6), 71.40 (C16), 69.67 (C15), 68.70 (C18), 67.77 (C13), 57.24 (C10), 55.47 (C19), 53.36 (C12) ppm; ^15^N NMR (^1^H^15^N HMBC, 61 MHz, D_2_O): *δ* = 331.2 (N5), 306.8 (N2), 47.7 (N11) ppm.

## [Pb(pazi)]

^1^H NMR (600 MHz, D_2_O): *δ* = 8.82 (s, 2H, H4), 8.62 (s, 2H, H6), 4.96 (d, ^2^*J* = 16.0 Hz, 2H, H10a), 4.28 (t, ^3^*J* = 11.1 Hz, 2H, H13a), 3.84 (dd, ^3^*J* = 14.2, ^3^*J* = 9.2 Hz, 4H, H10b, H18a), 3.70 (t, ^3^*J* = 10.3 Hz, 2H, H16a), 3.65 (t, ^3^*J* = 10.5 Hz, 2H, H15a), 3.59 (s, 2H, H15b), 3.57 – 3.54 (m, 2H, H13b), 3.44 (d, ^3^*J* = 10.4 Hz, 2H, H16b), 3.34 (d, ^3^*J* = 10.7 Hz, 2H, H18b), 3.19 (t, ^3^*J* = 12.7 Hz, 2H, H12a), 2.56 (d, ^3^*J* = 14.4 Hz, 2H, H12b), 2.43 (d, ^3^*J* = 10.4 Hz, 4H, H19) ppm; ^13^C NMR (151 MHz, D_2_O): *δ* = 169.97 (C7), 154.08 (C3), 147.38 (C4), 143.79 (C1), 143.32 (C6), 70.11 (C16), 69.43 (C15), 68.47 (C18), 67.25 (C13), 56.94 (C10), 54.76 (C19), 53.53 (C12) ppm; ^15^N NMR (^1^H^15^N HMBC, 61 MHz, D_2_O): *δ* = 329.8 (N5), 316.9 (N2), 47.7 (N11) ppm; ^207^Pb NMR (126 MHz, D_2_O): *δ* = -2223 ppm.

# NMR spectroscopy of [M(pym-mix)]^(n−2)+^ complexes

## [Ba(pym-mix)]

^1^H NMR (600 MHz, D_2_O): *δ* = 8.69 (d, ^3^*J* = 5.1 Hz, 1H), 8.54 (d, ^3^*J* = 5.2 Hz, 1H), 7.37 (d, ^3^*J* = 5.2 Hz, 1H), 7.35 (d, ^3^*J* = 5.1 Hz, 1H), 4.81 (d, ^3^*J* = 17.1 Hz, 1H, H10’a), 4.73 (d, ^2^*J* = 17.6 Hz, 1H, H10a), 4.18 (dd, ^2^*J* = 25.2, ^3^*J* = 12.2 Hz, 2H, H13a+13’a), 3.96 – 3.76 (m, 6H, H18a+18’a + H16a+16’a + H15a+15’a), 3.51 – 3.39 (m, 8H, H16b+16’b + H15b+15’b + H13b + 13’b, H10b + 10’b) 3.24 (p, ^3^*J* = 12.8 Hz, 4H, H12a + 12’a, H18b + 18’b), 2.62 (t, ^3^*J* = 13.2 Hz, 1H, H19a), 2.54 (d, ^3^*J* = 13.0 Hz, 1H, H19’a), 2.32 (dt, ^2^*J* = 23.6, ^3^*J* = 15.6 Hz, 3H, H12b+12’b + H19b), 2.22 (d, ^3^*J* = 14.2 Hz, 1H, H19’b) ppm; ^13^C NMR (151 MHz, D_2_O): *δ* = 169.01 (C1‘), 168.55 (C1), 168.40 (C3), 167.56 (C7‘), 159.70 (C6‘), 159.29 (C5), 159.20 (C7), 157.59 (C3‘), 121.41 (C4), 118.11 (C5‘), 70.29 (C16), 69.69 (C15), 67.44 (C18), 67.31 (C13), 67.21 (C13‘), 59.50 (C10‘), 57.91 (C10), 55.69 (C19), 55.33 (C19‘), 53.31 (C12‘), 53.12 (C12) ppm; ^15^N NMR (^1^H^15^N HMBC, 61 MHz, D_2_O): *δ* = 284.4 (N4‘), 283.9 (N6), 279.8 (N2), 279.2 (N2‘), 40.2 (N11‘) ppm.

## [La(pym-mix)]^+^

^1^H NMR (600 MHz, D_2_O): *δ* = 8.80 (d, ^3^*J* = 5.1 Hz, 1H, H6‘), 8.65 (dd, ^3^*J* = 5.2, 1.2 Hz, 1H, H5), 7.55 (dd, ^3^*J* = 5.2, ^4^*J* = 1.1 Hz, 1H, H4), 7.35 (d, ^3^*J* = 5.0 Hz, 1H, H5‘), 5.18 – 5.11 (m, 1H, H10‘a), 5.07 – 5.01 (m, 1H, H10a), 4.34 (ddd, ^3^*J* = 12.5, ^3^*J* = 10.4, ^4^*J* = 2.0 Hz, 1H, H13a), 4.29 (tt, ^3^*J* = 12.4, ^4^*J* = 2.0 Hz, 1H, H13‘a), 4.04 – 3.95 (m, 4H, H18a + H18’a + H16a + 16‘a), 3.88 (dtd, ^3^*J* = 14.6, ^3^*J* = 11.4, ^4^*J* = 2.6 Hz, 2H, H15a + H15‘a), 3.65 (ddd, ^3^*J* = 13.2, ^3^*J* = 7.3, ^4^*J* = 2.7 Hz, 2H, H13b + H13‘b), 3.59 – 3.49 (m, 6H, H10’b + H15b + H15’b + H10b + H16b + H16‘b), 3.39 (td, ^3^*J* = 13.3, ^4^*J* = 3.7 Hz, 2H, H12a), 3.37 – 3.31 (m, 2H, H18b + H18‘b), 2.68 – 2.63 (m, 1H, H12‘a), 2.63 – 2.56 (m, 2H, H19a + H19‘a), 2.56 – 2.51 (m, 1H, HH19b), 2.49 (d, ^3^*J* = 14.0 Hz, 1H, H12b), 2.38 (d, ^3^*J* = 14.1 Hz, 1H, H12‘b), 2.33 (d, ^3^*J* = 14.0 Hz, 1H, H19‘b) ppm; ^13^C NMR (151 MHz, D_2_O): *δ* = 169.72 (C1‘), 168.80 (C1), 168.24 (C3), 167.89 (C7‘), 161.51 (C6‘), 159.44 (C5), 157.20 (C7), 156.78 (C3‘), 122.36 (C4), 117.47 (C5‘), 71.37 (C16), 69.64 (C15), 68.61 (C18), 67.67 (C13), 67.58 (C13‘), 60.57 (C10‘), 58.95 (C10), 55.82 (C19), 55.61 (C19‘), 53.07 (C12‘), 52.94 (C12) ppm; ^15^N NMR (^1^H^15^N HMBC, 61 MHz, D_2_O): *δ* = 288.71 (N4‘), 281.11 (N6), 272.06 (N2), 269.69 (N2‘), 46.55 (N11), 46.28 (N11‘) ppm.

## [Pb(pym-mix)]

^1^H NMR (600 MHz, D_2_O): *δ* = 8.86 (d, ^3^*J* = 5.1 Hz, 1H. H6‘), 8.70 (d, ^3^*J* = 5.2 Hz, 1H, H5), 7.60 (d, ^3^*J* = 5.2 Hz, 1H, H4), 7.39 (d, ^3^*J* = 5.0 Hz, 1H, H5‘), 5.09 – 5.02 (m, 1H, H10‘a), 4.98 – 4.91 (m, 1H, H10a), 4.31 (t, ^3^*J* = 11.1 Hz, 1H, H13a), 4.25 (t, ^3^*J* = 11.1 Hz, 1H, H13‘a), 3.90 – 3.78 (m, 3H, H18a+18’a + H10‘b), 3.79 – 3.62 (m, 5H, H16a+16’a + H15a+15’a + H10b), 3.61 – 3.53 (m, 4H, H15b+15’b, H13b+13‘b), 3.49 – 3.44 (m, 2H, H16b+16‘b), 3.40 – 3.34 (m, 2H, H18b+18‘b), 3.17 (td, ^3^*J* = 13.2, ^4^*J* = 3.0 Hz, 2H, H12a+12‘a), 2.55 (d, ^3^*J* = 14.4 Hz, 1H, H12b), 2.50 (d, ^3^*J* = 14.8 Hz, 1H, H12‘b), 2.46 (d, ^3^*J* = 9.3 Hz, 3H, H19a+19’a, H19b), 2.40 (t, ^3^*J* = 12.5 Hz, 1H, H19‘b) ppm; ^13^C NMR (151 MHz, D_2_O): *δ* = 169.47 (C1‘), 168.79 (C1), 168.71 (C3), 162.83 (C7‘), 160.88 (C6‘), 158.76 (C5), 157.96 (C7), 157.23 (C3‘), 122.90 (C4), 118.60 (C5‘), 70.10 (C16), 69.45 (C15), 68.38 (C18), 67.29 (C13), 67.14 (C13‘), 60.28 (C10‘), 58.80 (C10), 55.24 (C19), 54.92 (C19‘), 53.23 (C12‘), 53.07 (C12); ^15^N NMR (^1^H^15^N HMBC, 61 MHz, D_2_O): *δ* = 287.9 (N4‘), 282.3 (N6), 281.2 (N2), 279.4 (N2‘), 46.6 (N11), 46.3 (N11‘) ppm; ^207^Pb (126 MHz, D_2_O): *δ* = -2281 ppm.

# ^1^H NMR spectra for p*K*_a_ determination

Figure S1. ^1^H NMR pD titration series of the new chelators: pym-2,4 (blue, top left), pym-2,6 (orange, top right), pym-mix (green, bottom left), and pazi (magenta, bottom right) obtained from the respective chelator (1 mM in 0.1 M NaCl aqueous D_2_O solution at (25 ± 1) °C) in the respective pD ranges indicated to the right side of each series. For clarity, only selected spectra and the spectral region from 2.4 to 9.2 ppm with a section cut from 5.4 to 7.0 are shown.

# ^13^C NMR spectra of the respective complexes

Figure S2. ^13^C NMR spectra of the complexes formed with the new chelators: pym-2,4 (blue, top left), pym-2,6 (orange, top right), pym-mix (green, bottom left), and pazi (magenta, bottom right) and the metal ions Ba^2+^, La^3+^ and Pb^2+^ obtained from 12.5 mM chelator and metal (1.2 eq.) in D_2_O at pH 6 (± 0.5) and 25 (± 1) °C.

# ^15^N NMR spectroscopy

**Table S4.** Measured ^15^N NMR chemical shifts of the amine nitrogen atoms of macropa (mcp) and the novel diaza chelators as well as of their metal complexes.

| **N_amine_** | **no metal pH 4 (pH 11)** | **Ba** | **Pb** | **La** |
| --- | --- | --- | --- | --- |
| **mcp** | 48 (39) | 42 | 47 | 48 |
| **pym-2,4** | 47 | 40 | 47 | 47 |
| **pym-2,6** | 46 | n.d.* | 47 | 47 |
| **pym-mix** | 46/46 | 40/46 | 47/46 | 47/46 |
| **pazi** | 47 | n.d.* | 48 | 48 |

* n.d., not detected

Table S5. Measured ^15^N NMR chemical shifts of the heteroaromatic nitrogen atoms in position 2 of macropa (mcp) and the novel diaza chelators as well as of their metal complexes.

| **N_arom_** | **no metal (pH 4)** | **Ba** | **Pb** | **La** |
| --- | --- | --- | --- | --- |
| **mcp** | 301 | 298 | 299 | 288 |
| **pym-2,4** | 279 | 278 | 280 | 270 |
| **pym-2,6** | 278 | 280 | 283 | 273 |
| **pym-mix** | 279/279 | 280/280 | 279/282 | 270/272 |
| **pazi** | 324 | 318 | 317 | 307 |

Table S6. Measured ^15^N NMR chemical shifts of the heteroaromatic nitrogen atoms in positions 4, 5, or 6 of the novel diaza chelators, respectively, as well as of their metal complexes.

| **N’_arom_** | **no metal (pH 4)** | **Ba** | **Pb** | **La** |
| --- | --- | --- | --- | --- |
| **pym-2,4** | 285 | 284 | 288 | 289 |
| **pym-2,6** | 282 | 279 | 283 | 281 |
| **pym-mix** | 285/282 | 284/279 | 288/281 | 289/281 |
| **pazi** | 326 | 324 | 330 | 331 |

# ^207^Pb NMR spectroscopy

Figure S3. ^207^Pb NMR spectra of Pb complexes with the pym-2,4, pym-2,6, pym-mix, pazi and macropa as well as of the Pb^2+^ aqua ion (in accordance with refs [^[2]^,^[3]^,^[4]^]), each in D_2_O solution at pD 6.

# HRMS spectra

Figure S4. HR-MS (ESI+) of the free chelator: macropa (top), pym-2,4 (middle) and pym-2,6 (bottom).

Figure S5. HR-MS (ESI+) of the free chelator: pym‑mix (top) and pazi (bottom).

Figure S6. HR-MS (ESI+) of the complexes: [Ba(pym-2,4)] (top), [La(pym-2,4)]^+^ (middle) and [Pb(pym-2,4)] (bottom).

Figure S7. HR-MS (ESI+) of the complexes: [Ba(pym-2,6)] (top), [La(pym-2,6)]^+^ (middle) and [Pb(pym-2,6)] (bottom).

Figure S8. HR-MS (ESI+) of the complexes: [Ba(pazi)] (top), [La(pazi)]^+^ (middle) and [Pb(pazi)] (bottom).

Figure S9. HR-MS (ESI+) of the complexes: [Ba(pym-mix)] (top), [La(pym-mix)]^+^ (middle) and [Pb(pym-mix)] (bottom).

# Isothermal Titration Calorimetry (ITC)

A *MicroCal* PEAQ-ITC (Malvern Panalytical) calorimeter (total volume 200 µL) was used for calorimetric measurements. The titrant (40 μL) was injected into the sample cell at 25 °C by an automated syringe under continuous stirring (750 rpm). The titration was conducted in 19 injections of 2 μL each (0.5 µL for the first injection) with an injection spacing of 150 s (Ba, Pb) or 600 s (La). The syringe was filled with the chelator in concentrations of 600 μM or 6 mM using a 0.1 M NaCl solution, and the sample cell contained 30, 50, and 100 μM (Ba, Pb) or 300, 500, and 1000 μM (La) metal in a 0.1 M NaCl solution at the same specific pH (± 0.1). An analogous setup without metal was used as the background measurement. ITC data were analyzed using a MATLAB code based on speciation calculations^[5]^ and equations (e.g., displaced volume) provided by Malvern. The estimation of errors and the goodness of the model was performed with a Monte Carlo approach as previously described.^[6]^

Preparation of the solution for ITC measurements:

Using stock metal salt and chelator solutions with concentrations of 1 mM. The desired concentrations were pipetted and the respected pH was set: Ba (pH 5), Pb (pH 2), La (pH 5).

Table S7. Parameters of ITC measurements.

| Temperature (°C) | | | 25 | | |
| --- | --- | --- | --- | --- | --- |
| Reference power (µcal/s) | | | 7.00 | | |
| FeedBack | | | High | | |
| Stirring speed (rpm) | | | 750 | | |
| Initial delay (s) | | | 60 | | |
| Number of injections | | | 19 | | |
| Concentration syringe (µM) | | | 600 (Ba, Pb) or 4800 (La) | | |
| concentration cell (µM) | | | 30, 50, 100 (Ba, Pb) or 250, 400, 800 (La) | | |
|  | | |  | | |
| Injection | Volume (µL) | Duration (s) | | Spacing (s) |  |
| 1 | 0.4 | 0.8 | | 150 (Ba, Pb) or 600 (La) |  |
| 2 – 19 | 2.0 | 4.0 | | 150 (Ba, Pb) or 600 (La) |  |

Table S8. Results of the ITC measurements.

| Chelator | metal | n | error | log *β* | error | ΔH | error |
| --- | --- | --- | --- | --- | --- | --- | --- |
| **pym-2,4** | Ba | 0.99 | 0.04 | 10.07 | 0.11 | -39.36 | 2.49 |
|  | Pb | 0.95 | 0.02 | 15.48 | 0.16 | -44.87 | 1.31 |
|  | La | 0.91 | 0.05 | 11.76 | 0.17 | 8.42 | 0.38 |
| **pym-2,6** | Ba | 0.85 | 0.03 | 10.18 | 0.08 | -40.65 | 2.03 |
|  | Pb | 0.86 | 0.03 | 15.28 | 0.16 | -47.29 | 1.72 |
|  | La | 0.97 | 0.07 | 12.56 | 0.38 | 6.64 | 0.41 |
| **pazi** | Ba | 1.09 | 0.09 | 10.07 | 0.27 | -33.51 | 2.65 |
|  | Pb | 1.09 | 0.04 | 15.44 | 0.22 | -42.52 | 2.92 |
|  | La | 0.91 | 0.03 | 11.85 | 0.05 | 13.42 | 0.30 |
| **pym-mix** | Ba | 1.09 | 0.04 | 10.32 | 0.16 | -43.70 | 1.55 |
|  | Pb | 1.09 | 0.04 | 15.50 | 0.06 | -43.74 | 1.55 |
|  | La | 0.89 | 0,05 | 12.03 | 0.05 | 7.23 | 0.16 |

## [Ba(pym-2,4)]

**Figure S10.** ITC data of the three measurements of 600 µM **pym-2,4** to different concentrations (30–100 µM) of BaCl_2_ solution in 100 mM NaCl.

## [Pb(pym-2,4)]

**Figure S11.** ITC data of the three measurements of 600 µM **pym-2,4** to different concentrations (30–100 µM) of Pb(NO_3_)_2_ solution in 100 mM NaCl.

## [Ba(pym-2,6)]

**Figure S12.** ITC data of the three measurements of 600 µM **pym-2,6** to different concentrations (30–100 µM) of BaCl_2_ solution in 100 mM NaCl.

## [Pb(pym-2,6)]

**Figure S13.** ITC data of the three measurements of 600 µM **pym-2,6** to different concentrations (30–100 µM) of Pb(NO_3_)_2_ solution in 100 mM NaCl.

## [Ba(pazi)]

**Figure S14.** ITC data of the three measurements of 600 µM **pazi** to different concentrations (30–100 µM) of BaCl_2_ solution in 100 mM NaCl.

## [Pb(pazi)]

**Figure S15.** ITC data of the three measurements of 600 µM **pazi** to different concentrations (30–100 µM) of Pb(NO_3_)_2_ solution in 100 mM NaCl.

## [Ba(pym-mix)]

**Figure S16.** ITC data of the three measurements of 600 µM **pym-mix** to different concentrations (30–100 µM) of BaCl_2_ solution in 100 mM NaCl.

## [Pb(pym-mix)]

**Figure S17.** ITC data of the three measurements of 600 µM **pym-mix** to different concentrations (30–100 µM) of Pb(NO_3_)_2_ solution in 100 mM NaCl.

## La-complexes data processing

As described in Blei *et al.*^[7]^ a small rise in the curve was observed at the beginning of the integrated thermograms. This was previously determined by the assumption of La-complex formation in a 2:1 ratio. However, subsequent investigations indicated that the observed effect was more likely to be attributable to the pH shift that occurred during the titration process. This shift was characterized by a total change from a pH of 5 to a pH of 3.6. This pH shift may be the cause of the rising curve that is initially observed in the titration. As an indirect proof, a titration was conducted in a strong buffer at a specific pH value, the results of which are presented below.

Figure S18: ITC results for a titration of 600 µM pym-2,4 to 50 µM La(NO_3_)_3_ in 10 mM 2-hydroxy-3-morpholinopropanesulfonic acid (MOPSO) buffer at pH 6.6.

In this instance, the initial rise is not observed, which suggests that the observation is more influenced by the pH level rather than the formation of a 2:1 complex. However, this configuration is not appropriate for thermodynamic calculations to determine complex stability constants (log *β*) because the resulting curve is too steep. The titrations were conducted at a pH of 5, with the absence of buffers, and it was evaluated and processed as s 1:1 complex forming.

## [La(pym-2,4)]

**Figure S19.** ITC data of the three measurements of 600 µM **pym-2,4** to different concentrations (30–100 µM) of La(NO_3_)_3_ solution in 100 mM NaCl.

## [La(pym-2,6)]

**Figure S20.** ITC data of the three measurements of 600 µM **pym-2,6** to different concentrations (30–100 µM) of La(NO_3_)_3_ solution in 100 mM NaCl.

## [La(pazi)]

**Figure S21.** ITC data of the three measurements of 600 µM **pazi** to different concentrations (30–100 µM) of La(NO_3_)_3_ solution in 100 mM NaCl.

## [La(pym-mix)]

**Figure S22.** ITC data of the three measurements of 600 µM **pym-mix** to different concentrations (30–100 µM) of La(NO_3_)_3_ solution in 100 mM NaCl.

# Radiolabeling

*CAUTION! The selected radionuclides ^131^Ba, ^133^La, ^203^Pb, ^212^Pb, ^223^Ra, ^224^Ra, and ^225^Ac represent α, β, and γ emitting radionuclides. Therefore, the following experiments were performed in a controlled area where the handling of radioactive materials is permitted and regulated.*

The radiometals ^131^Ba and ^133^La were in-house produced by cyclotron-assisted proton irradiation of stable CsCl and enriched [^134^Ba]BaCO_3_, respectively, using a TR-FLEX cyclotron (ACSI). The radiochemical processing of the irradiated targets is described elsewhere.^22,39^ ^131^Ba was formulated as [^131^Ba]Ba(NO_3_)_2_ in 0.05 M HNO_3_ while ^133^La as [^133^La]LaCl_3_ in 0.05 M HCl aqueous solution. Moreover, ^223^Ra was used from a Xofigo® solution. ^225^Ac was purchased from ITM Munich (Germany) as a 0.1 M HCl solution and used without further puriﬁcation. ^203^Pb and ^224^Ra were provided through PRISMAP as NH_4_OAc buffer (pH 5 − 7) and chloride solution, respectively. Furthermore, ^212^Pb was isolated from its parent nuclide ^224^Ra in an inverse generator based on a crown-ether chromatography resin. Briefly, 180 mg of TK102 (TrisKem, Bruz, France) were packed into an empty 2 mL column and conditioned with 3 × 0.5 mL of H_2_O and 3 × 0.5 mL of 1 M HCl. The [^224^Ra]RaCl_2_ was loaded onto this column, washed with 2 × 0.5 mL of 1 M HCl to quantitively recover the ^224^Ra, followed by 6 × 0.5 mL of 1 M HCl to remove the remaining radium traces. The ^212^Pb was then eluted with 4 × 0.5 mL of 1 M NH_4_OAc and directly used for radiolabeling. Radiolabeling was carried out to demonstrate the applicability of the novel chelators for radiopharmaceutical purposes with different radionuclides. Therefore, 100 kBq of ^203^Pb, ^212^Pb, ^133^La, ^225^Ac, and ^131^Ba as well as 10 kBq of ^223^Ra were incubated with different concentrations (10^−3^, 10^−4^, 10^−5^, 10^−6^, 10^−7^, 5∙10^−8^ M) of the respective chelator in 0.2 M NH_4_OAc buffer at pH 6 for 1 h at 25 °C. Two TLC systems, normal-phase (NP-TLC) and reversed-phase (RP-TLC), were used to monitor the radiochemical conversion (RCC).^32,39^ The first system (RP-TLC) was designed to quantify the RCC of the radiolabeling reaction and employed an RP-18 TLC plate with an acetonitrile/water (7:3) mobile phase. The second system (NP-TLC) was used to assess complex stability in a competition experiment with EDTA. In this setup, a normal-phase TLC plate and a 50 mM EDTA solution at pH 6 served as the mobile phase.

The ^223^Ra complexes (10^–3^ M, 5 kBq, 50 µL) were diluted in PBS (150 µL) and human serum (250 µL) and stirred at 37°C over 15 days (360 h). The NP-TLC system was used for evaluation (eluent: 50 mM EDTA, pH 6).

Table S9. Results of radiochemical conversion (RCC) obtained with ^133^La and ^225^Ac for the two TLC systems: the first system was a mixture of 7:3 acetonitrile/water (ACN/H_2_O) on a reverse-phase TLC and the second system was a 50 mM ethylenediaminetetraacetic acid (EDTA) at pH 6 on a normal-phase TLC.

| **^133^La** | **mcp** | | | | **pym-2,4** | | | | **pym-2,6** | | | | **pym-mix** | | | | **pazi** | | | |
| --- | --- | --- | --- | --- | --- | --- | --- | --- | --- | --- | --- | --- | --- | --- | --- | --- | --- | --- | --- | --- |
|  | H_2_O/ACN | | ETDA | | H_2_O/ACN | | ETDA | | H_2_O/ACN | | ETDA | | H_2_O/ACN | | ETDA | | H_2_O/ACN | | ETDA | |
| M (chelator) | RCC | error | RCC | error | RCC | error | RCC | error | RCC | error | RCC | error | RCC | error | RCC | error | RCC | error | RCC | error |
| 10^‒3^ | >99 | n/a | >99 | n/a | >99 | n/a | >99 | n/a | >99 | n/a | >99 | n/a | >99 | n/a | >99 | n/a | >99 | n/a | >99 | n/a |
| 10^‒4^ | >99 | n/a | >99 | n/a | >99 | n/a | >99 | n/a | >99 | n/a | >99 | n/a | >99 | n/a | >99 | n/a | >99 | n/a | >99 | n/a |
| 10^‒5^ | >99 | n/a | >99 | n/a | >99 | n/a | >99 | n/a | >99 | n/a | >99 | n/a | 98.8 | 0.5 | >99 | n/a | >99 | n/a | >99 | n/a |
| 10^‒6^ | >99 | n/a | >99 | n/a | 97.5 | 2.4 | >99 | n/a | 96.3 | 3.2 | >99 | n/a | 97.3 | 2.2 | >99 | n/a | 98.3 | 1.0 | >99 | n/a |
| 10^‒7^ | 63.7 | 28.0 | 73.7 | 30.1 | 97.3 | 2.9 | 79.0 | 34.6 | 85.7 | 16.7 | 93.7 | 9.2 | 66.3 | 30.7 | 74.5 | 21.2 | 37.0 | 5.6 | 70.7 | 23.2 |
|  |  |  |  |  |  |  |  |  |  |  |  |  |  |  |  |  |  |  |  |  |
| **^225^Ac** | **mcp** | | | | **pym-2,4** | | | | **pym-2,6** | | | | **pym-mix** | | | | **pazi** | | | |
|  | H_2_O/ACN | | ETDA | | H_2_O/ACN | | ETDA | | H_2_O/ACN | | ETDA | | H_2_O/ACN | | ETDA | | H_2_O/ACN | | ETDA | |
| M (chelator) | RCC | error | RCC | error | RCC | error | RCC | error | RCC | error | RCC | error | RCC | error | RCC | error | RCC | error | RCC | error |
| 10^‒3^ | >99 | n/a | >99 | n/a | >99 | n/a | >99 | n/a | >99 | n/a | >99 | n/a | >99 | n/a | >99 | n/a | >99 | n/a | >99 | n/a |
| 10^‒4^ | >99 | n/a | >99 | n/a | >99 | n/a | >99 | n/a | >99 | n/a | >99 | n/a | >99 | n/a | >99 | n/a | >99 | n/a | >99 | n/a |
| 10^‒5^ | >99 | n/a | >99 | n/a | >99 | n/a | >99 | n/a | >99 | n/a | >99 | n/a | >99 | n/a | >99 | n/a | >99 | n/a | >99 | n/a |
| 10^‒6^ | 92.9 | 5.5 | 99.0 | 1.3 | 93.1 | 5.5 | 98.1 | 2.6 | 98.1 | 1.6 | >99 | n/a | 93.3 | 7.4 | 96.5 | 4.9 | 98.7 | 1.2 | >99 | n/a |
| 10^‒7^ | 2.8 | 1.8 | 0.6 | 0.4 | 0.3 | 0.3 | 0.7 | 0.7 | 2.0 | 1.4 | 1.8 | 1.4 | 0.7 | 0.2 | 0.5 | 0.4 | 2.1 | 1.6 | 1.6 | 1.6 |

Table S10. Results of radiochemical conversion (RCC) obtained with ^203^Pb and ^212^Pb for the two TLC systems: the first system was a mixture of 7:3 acetonitrile/water (H_2_O/ACN) on a reverse-phase TLC and the second system was a 50 mM ethylenediaminetetraacetic acid (EDTA) at pH 6 on a normal-phase TLC.

| **^203^Pb** | **mcp** | | | | **pym-2,4** | | | | **pym-2,6** | | | | **pym-mix** | | | | **pazi** | | | |
| --- | --- | --- | --- | --- | --- | --- | --- | --- | --- | --- | --- | --- | --- | --- | --- | --- | --- | --- | --- | --- |
|  | H_2_O/ACN | | ETDA | | H_2_O/ACN | | ETDA | | H_2_O/ACN | | ETDA | | H_2_O/ACN | | ETDA | | H_2_O/ACN | | ETDA | |
| M (chelator) | RCC | error | RCC | error | RCC | error | RCC | error | RCC | error | RCC | error | RCC | error | RCC | error | RCC | error | RCC | error |
| 10^‒4^ | >99 | n/a | >99 | n/a | >99 | n/a | >99 | n/a | >99 | n/a | >99 | n/a | >99 | n/a | >99 | n/a | >99 | n/a | >99 | n/a |
| 10^‒5^ | >99 | n/a | >99 | n/a | 98.6 | 1.3 | >99 | n/a | 76.6 | 9.2 | >99 | n/a | 94.9 | 3.8 | >99 | n/a | 98.1 | 1.1 | >99 | n/a |
| 10^‒6^ | 90.1 | 11.5 | 97.3 | 3.2 | 91.1 | 7.6 | 96.7 | 3.2 | 65.9 | 19.3 | 96.0 | 2.0 | 90.1 | 5.4 | 98.4 | 1.8 | 95.3 | 4.1 | 97.2 | 3.9 |
| 10^‒7^ | 21.1 | 13.1 | 25.6 | 16.7 | 17.8 | 18.7 | 42.2 | 28.3 | 5.3 | 2.6 | 6.8 | 3.6 | 12.9 | 5.7 | 18.3 | 10.5 | 27.9 | 17.0 | 49.3 | 13.0 |
| 5× 10^‒8^ | 5.1 | 6.8 | 5.7 | 5.0 | 1.6 | 0.5 | 5.6 | 2.9 | 4.3 | 3.0 | 0.1 | 0.1 | 6.8 | 3.1 | 8.5 | 6.8 | 9.2 | 2.3 | 20.5 | 14.7 |
|  |  |  |  |  |  |  |  |  |  |  |  |  |  |  |  |  |  |  |  |  |
| **^212^Pb** | **mcp** | | | | **pym-2,4** | | | | **pym-2,6** | | | | **pym-mix** | | | | **pazi** | | | |
|  | H_2_O/ACN | | ETDA | | H_2_O/ACN | | ETDA | | H_2_O/ACN | | ETDA | | H_2_O/ACN | | ETDA | | H_2_O/ACN | | ETDA | |
| M (chelator) | RCC | error | RCC | error | RCC | error | RCC | error | RCC | error | RCC | error | RCC | error | RCC | error | RCC | error | RCC | error |
| 10^‒4^ | >99 | n/a | >99 | n/a | >99 | n/a | >99 | n/a | >99 | n/a | >99 | n/a | >99 | n/a | >99 | n/a | >99 | n/a | >99 | n/a |
| 10^‒5^ | >99 | n/a | >99 | n/a | >99 | n/a | >99 | n/a | 98.7 | 0.4 | >99 | n/a | >99 | n/a | >99 | n/a | >99 | n/a | >99 | n/a |
| 10^‒6^ | >99 | n/a | >99 | n/a | >99 | n/a | >99 | n/a | 98.4 | 0.9 | >99 | n/a | >99 | n/a | >99 | n/a | >99 | n/a | >99 | n/a |
| 10^‒7^ | 95.9 | 6.0 | 96.0 | 2.6 | >99 | n/a | 91.0 | 14.1 | 74.0 | 3.6 | 89.6 | 8.7 | >99 | n/a | >99 | n/a | >99 | n/a | >99 | n/a |
| 5× 10^‒8^ | 29.0 | 2.8 | 60.5 | 3.5 | 14.5 | 4.9 | 39.5 | 22.5 | 4.5 | 3.5 | 12.5 | 7.8 | 14.0 | 9.9 | 38.0 | 12.7 | 39.0 | 23.5 | 44.3 | 11.6 |

Table S11. Results of radiochemical conversion (RCC) obtained with ^131^Ba and ^223^Ra for the two TLC systems: the first system was a mixture of 7:3 acetonitrile/water (H_2_O/ACN) on a reverse-phase TLC and the second system was a 50 mM ethylenediaminetetraacetic acid (EDTA) at pH 6 on a normal-phase TLC. Additionally, the ^223^Ra-labeled complexes and free [^223^Ra]Ra^2+^ were incubated in human serum and the RCC was observed after selective time points.

| **^131^Ba** | **mcp** | | | | | **pym-2,4** | | | | | | **pym-2,6** | | | | | **pym-mix** | | | | | **pazi** | | | | |
| --- | --- | --- | --- | --- | --- | --- | --- | --- | --- | --- | --- | --- | --- | --- | --- | --- | --- | --- | --- | --- | --- | --- | --- | --- | --- | --- |
|  | H_2_O/ACN | | | ETDA | | H_2_O/ACN | | | ETDA | | | H_2_O/ACN | | ETDA | | | H_2_O/ACN | | | ETDA | | H_2_O/ACN | | | ETDA | |
| M (chelator) | RCC | error | | RCC | error | RCC | | error | RCC | error | | RCC | error | RCC | | error | RCC | error | | RCC | error | RCC | | error | RCC | error |
| 10^‒3^ | >99 | n/a | | 94.7 | 4.0 | >99 | | n/a | >99 | n/a | | >99 | n/a | >99 | | n/a | >99 | n/a | | >99 | n/a | >99 | | n/a | >99 | n/a |
| 10^‒4^ | 97.5 | 3.0 | | 62.3 | 11.0 | >99 | | n/a | >99 | n/a | | >99 | n/a | >99 | | n/a | >99 | n/a | | >99 | n/a | >99 | | n/a | >99 | n/a |
| 10^‒5^ | 89.8 | 4.1 | | 23.7 | 3.5 | 98.8 | | 0.4 | 92.5 | 4.6 | | 95.5 | 4.6 | 92.5 | | 0.5 | 99.0 | 1.0 | | 99.0 | 1.0 | 98.3 | | 1.3 | 94.0 | 2.8 |
| 10^‒6^ | 74.5 | 2.1 | | 12.3 | 6.4 | 95.3 | | 4.3 | 90.3 | 4.8 | | 93.0 | 4.6 | 90.3 | | 2.4 | 93.3 | 3.3 | | 93.8 | 3.3 | 90.0 | | 6.4 | 92.3 | 3.6 |
| 10^‒7^ | 9.0 | 5.7 | | 4.6 | 2.1 | 93.0 | | 4.3 | 89.5 | 5.3 | | 85.5 | 2.7 | 89.5 | | 2.7 | 77.0 | 8.6 | | 75.5 | 8.7 | 79.5 | | 8.0 | 85.0 | 4.1 |
|  |  |  | |  |  |  | |  |  |  | |  |  |  | |  |  |  | |  |  |  | |  |  |  |
| **^223^Ra** | **mcp** | | | | | **pym-2,4** | | | | | | **pym-2,6** | | | | | **pym-mix** | | | | | **pazi** | | | | |
|  | H_2_O/ACN | | | ETDA | | H_2_O/ACN | | | ETDA | | | H_2_O/ACN | | ETDA | | | H_2_O/ACN | | | ETDA | | H_2_O/ACN | | | ETDA | |
| M (chelator) | RCC | error | | RCC | error | RCC | | error | RCC | error | | RCC | error | RCC | | error | RCC | error | | RCC | error | RCC | | error | RCC | error |
| 10^‒3^ |  |  | | 72.7 | 6.2 |  | |  | >99 | n/a | |  |  | >99 | | n/a |  |  | | >99 | n/a |  | |  | >99 | n/a |
| 10^‒4^ |  |  | | 35.8 | 13.5 |  | |  | 95.2 | 5.2 | |  |  | 98.2 | | 1.3 |  |  | | 97.3 | 1.7 |  | |  | 95.9 | 3.6 |
| 10^‒5^ |  |  | | 2.0 | 2.1 |  | |  | 49.4 | 17.7 | |  |  | 44.0 | | 10.7 |  |  | | 44.0 | 9.2 |  | |  | 46.3 | 23.6 |
|  |  |  | |  |  |  | |  |  |  | |  |  |  | |  |  |  | |  |  |  | |  |  |  |
|  |  |  | |  |  |  | |  |  |  | |  |  |  | |  |  |  | |  |  |  | |  |  |  |
|  |  |  | |  |  |  | |  |  |  | |  |  |  | |  |  |  | |  |  |  | |  |  |  |
|  |  |  | |  |  |  | |  |  |  | |  |  |  | |  |  |  | |  |  |  | |  |  |  |
| **Table S12.** Stability of the ^223^Ra complexes in human serum. | | | | | | | | | | | | | | | | | | | | | | | | | | |
| **t in h** | | | **blank** | | | | **mcp** | | | | **pym-2,4** | | | | **pym-2,6** | | | | **pym-mix** | | | | **pazi** | | | |
| 0 | | | 3.0 | | | | 80.8 | | | | 99.5 | | | | 99.9 | | | | 99.9 | | | | 99.9 | | | |
| 1 | | | 4.6 | | | | 85.6 | | | | 96.3 | | | | 99.0 | | | | 97.1 | | | | 99.9 | | | |
| 24 | | | 4.6 | | | | 79.2 | | | | 99.6 | | | | 99.2 | | | | 98.8 | | | | 99.0 | | | |
| 48 | | | 4.6 | | | | 68.4 | | | | 94.9 | | | | 98.0 | | | | 97.2 | | | | 98.4 | | | |
| 120 | | | 0.0 | | | | 43.3 | | | | 94.2 | | | | 96.9 | | | | 95.9 | | | | 98.4 | | | |
| 192 | | | 0.8 | | | | 4.6 | | | | 94.2 | | | | 96.9 | | | | 94.7 | | | | 99.5 | | | |
| 336 | | | 0.5 | | | | 4.6 | | | | 92.7 | | | | 97.2 | | | | 85.2 | | | | 97.3 | | | |

# Computational Calculations

Geometry optimizations of the 1:1 complexes of Ba^2+^, La^3+^, Pb^2+^, Ac^3+^ and Ra^2+^ with the chelators were performed in Turbomole 7.3.1 using Density Functional Theory (DFT).^[8]^ To reflect known crystal structures of the macropa chelator, a single water molecule was included within the coordination sphere, except for Pb^2+^, where no additional water molecule is expected in the first coordination sphere.^32,49^ The resulting input complexes exhibited a coordination number of 11, consisting of 10 bonds from the modified macropa chelator (N_4_O_6_ donor set) to the metal center and one from the coordinated water molecule. Solvent effects were incorporated using the conductor-like screening model (COSMO) with implicit water solvation (dielectric constant ε = 80.3).^[9]^ The PBE0 hybrid functional was employed in conjunction with the def-TZVP basis set and an effective core potential (Ba, La: ECP-46 Pb, Ac: ECP-60, Ra: ECP-78).^[10],^^[11],^^[12],^^[13],^^[14],^^[15],^^[16]^ Additionally, Grimme’s DFT-D3 dispersion correction was applied to account for dispersion interactions.^[17]^ To ensure convergence to a local energy minimum of the optimized structures, a numerical vibrational frequency analysis was performed, confirming the absence of imaginary frequencies. Structural visualization and analysis of the optimized geometries were carried out using ChimeraX.^XVII^ NMR shielding constants were computed using the mpshift module in Turbomole.

## Determination of the bond lengths M‒N_arom_

Figure S23. The bond lengths d in Å of the donor atoms N_arom_ (aromatic nitrogen atom) of macropa (gray), pym-2,4 (blue), pym-2,6 (orange), pym-mix (green), and pazi (magenta) forming complexes with the metal ions Pb^2+^, La^3+^, Ac^3+^, Ba^2+^, and Ra^2+^.

## Determination of the bond lengths M‒O_carbox_

Figure S24. The bond lengths d in Å of the donor atoms O_carbox_ (carboxylic oxygen atom) of macropa (gray), pym-2,4 (blue), pym-2,6 (orange), pym-mix (green), and pazi (magenta) forming complexes with the metal ions Pb^2+^, La^3+^, Ac^3+^, Ba^2+^, and Ra^2+^.

## Determination of the bond lengths M‒N_amine_

Figure S25. The bond lengths d in Å of the donor atoms N_amine_ (amine nitrogen atom) of macropa (gray), pym-2,4 (blue), pym-2,6 (orange), pym-mix (green), and pazi (magenta) forming complexes with the metal ions Pb^2+^, La^3+^, Ac^3+^, Ba^2+^, and Ra^2+^.

## Determination of the bond lengths M‒O_macro_

Figure S26. The bond lengths d in Å of the donor atoms O_macro_ (ether oxygen) of macropa (gray), pym-2,4 (blue), pym-2,6 (orange), pym-mix (green), and pazi (magenta) forming complexes with the metal ions Pb^2+^, La^3+^, Ac^3+^, Ba^2+^, and Ra^2+^.

## Molecular structures of the computed complexes

Figure S27. Side and top views of geometry-optimized [M(pym-2,6)(H_2_O)]^(m−2)+^, [M(pym-mix)(H_2_O)]^(m−2)+^ and [M(pazi)(H_2_O)]^(m−2)+^ complexes (M = Ra^2+^, Ba^2+^, La^3+^, and Ac^3+^) and [Pb(pym/pazi)] complexes in aqueous solutions.

1. Friedrich, S.; Näder, A.; Drobot, B.; Kretzschmar, J.; Stumpf, T.; Barkleit, A. Synthesis of Nonadentate Ligand Diethylene Glycol-Bis(3-Aminopropyl Ether)-*N,N,N′,N′*-Tetraacetic Acid DEGTA and Its Complexation Behavior toward Trivalent Lanthanides and Actinides. *Inorg. Chem.*, **2025**, *64*, 5014.
2. G. E. Maciel, J. L. Dallas, *J. Am. Chem. Soc.* **1973**, *95*, 3039.
3. P. G. Harrison, M. A. Healy, A. T. Steel, *J. Chem. Soc. Dalton Trans.* **1983**, 1845.
4. G. M. Bernard, V. K. Michaelis, *Magn. Reson. Chem.* **2020**, *58*, 1203.
5. Smith, D. S. Solution of Simultaneous Chemical Equilibria in Heterogeneous Systems: Implementation in Matlab; Chemistry Faculty Publications, 2019; p 14; https://scholars.wlu.ca/chem_faculty/14
6. Drobot, B.; Schmidt, M.; Mochizuki, Y.; Abe, T.; Okuwaki, K.; Brulfert, F.; Falke, S.; Samsonov, S.; Komeiji, Y.; Betzel, C.; Stumpf, T.; Raff, J.; Tsushima, S. Cm^3+^/Eu^3+^ induced structural, mechanistic and functional implications for calmodulin. *Phys. Chem., Chem. Phys.* **2019**, *21*, 21213
7. M. K. Blei, L. Waurick, F. Reissig, K. Kopka, T. Stumpf, B. Drobot, J. Kretzschmar, C. Mamat, *Inorg. Chem.* **2023**, *62*, 20699.
8. TURBOMOLE V7.3 2018, a development of University of Karlsruhe and Forschungszentrum Karlsruhe GmbH, 1989-2007, TURBOMOLE GmbH, since 2007; available from https://www.turbomole.org
9. Klamt, A.; Schüürmann, G. COSMO: A new approach to dielectric screening in solvents with explicit expressions for the screening energy and its gradient. *J. Chem. Soc. Perkin Trans. 2* **1993**, *5*, 799.
10. Perdew, J. P.; Emzerhof, M.; Burke, K., Rationale for mixing exact exchange with density functional approximations, J. Chem. Phys. 1996, 105, 9982.
11. Adamo C.; Barone, V. Toward reliable density functional methods without adjustable parameters: The PBE0 model. *J. Chem. Phys.* **1999**, *110*, 6158.
12. Lim, I.; Stoll, H.; Schwerdtfeger, P. Relativistic small-core energy-consistent pseudopotentials for the alkaline-earth elements from Ca to Ra. *J. Chem. Phys.* **2006**, *124*, 034107.
13. Cao X.; Dolg, M. Segmented contraction scheme for small-core actinide pseudopotential basis sets. *J. Mol. Struct.* **2004**, *673*, 203.
14. Gulde, R.; Pollak, P.; Weigend, F. Error-Balanced Segmented Contracted Basis Sets of Double-ζ to Quadruple-ζ Valence Quality for the Lanthanides. *J. Chem. Theory Comp.* **2012**, *8*, 4062.
15. Weigend, F.; Ahlrichs, R. Balanced basis sets of split valence, triple zeta valence and quadruple zeta valence quality for H to Rn: Design and assessment of accuracy. *Phys. Chem. Chem. Phys.* **2005**, *7*, 3297.
16. Grimme, S.; Antony, J.; Ehrlich, S.; Krieg, H. A consistent and accurate ab initio parametrization of density functional dispersion correction (DFT-D) for the 94 elements H-Pu. *J. Chem. Phys.* **2010**, *132*, 154104.
17. Meng, E. C.; Goddard, T. D.; Pettersen, E. F.; Couch, G. S.; Pearson, Z. J.; Morris, J. H.; Ferrin, T. E. UCSF ChimeraX: Tools for structure building and analysis. *Protein Sci.* **2023**, *32*, e4792.
